# Supplementary material for: Protocol for the SAFEST review: the Shock-Absorbing Flooring Effectiveness SysTematic review including older adults and staff in hospitals and care homes
Source: BMJ Open. 2020 Feb 17;10(2):e032315. doi: 10.1136/bmjopen-2019-032315 (PMC7044972; doi:10.1136/bmjopen-2019-032315)
Supplement: Supplementary data [file bmjopen-2019-032315supp001.pdf]

PRISMA- P 2015 Checklist

This checklist has been adapted from the operationalized checklist available from: http://www.prisma-statement.org/Extensions/Protocols.aspx which is based on Table 3 in Moher et al. Syst Rev. 2015;4(1):1. doi: 10.1186/2046-4053-4-1

Section/topic	#	Checklist item	Information reported		Page number(s)
			Yes	No	
**ADMINISTRATIVE INFORMATION**					
**Title**					
Identification	1a	Identify the report as a protocol of a systematic review	☒	☐	1
Update	1b	If the protocol is for an update of a previous systematic review, identify as such	☐	☒	n/a
**Registration**	2	If registered, provide the name of the registry (e.g., PROSPERO) and registration number in the Abstract	☒	☐	2
**Authors**					
Contact	3a	Provide name, institutional affiliation, and e-mail address of all protocol authors; provide physical mailing address of corresponding author	☒	☐	1
Contributions	3b	Describe contributions of protocol authors and identify the guarantor of the review	☒	☐	22
**Amendments**	4	If the protocol represents an amendment of a previously completed or published protocol, identify as such and list changes; otherwise, state plan for documenting important protocol amendments	☒	☐	5
**Support**					
Sources	5a	Indicate sources of financial or other support for the review	☒	☐	22
Sponsor	5b	Provide name for the review funder and/or sponsor	☒	☐	22
Role of sponsor/funder	5c	Describe roles of funder(s), sponsor(s), and/or institution(s), if any, in developing the protocol	☒	☐	22
**INTRODUCTION**					
**Rationale**	6	Describe the rationale for the review in the context of what is already known	☒	☐	4
**Objectives**	7	Provide an explicit statement of the question(s) the review will address with reference to participants, interventions, comparators, and outcomes (PICO)	☒	☐	5
**METHODS**					

Section/topic	#	Checklist item	Information reported		Page number(s)
			Yes	No	
**Eligibility criteria**	8	Specify the study characteristics (e.g., PICO, study design, setting, time frame) and report characteristics (e.g., years considered, language, publication status) to be used as criteria for eligibility for the review	☒	☐	5-7
**Information sources**	9	Describe all intended information sources (e.g., electronic databases, contact with study authors, trial registers, or other grey literature sources) with planned dates of coverage	☒	☐	7-8
**Search strategy**	10	Present draft of search strategy to be used for at least one electronic database, including planned limits, such that it could be repeated	☒	☐	Supplementary file 2
STUDY RECORDS					
Data management	11a	Describe the mechanism(s) that will be used to manage records and data throughout the review	☒	☐	8
Selection process	11b	State the process that will be used for selecting studies (e.g., two independent reviewers) through each phase of the review (i.e., screening, eligibility, and inclusion in meta-analysis)	☒	☐	9
Data collection process	11c	Describe planned method of extracting data from reports (e.g., piloting forms, done independently, in duplicate), any processes for obtaining and confirming data from investigators	☒	☐	9,11
**Data items**	12	List and define all variables for which data will be sought (e.g., PICO items, funding sources), any pre-planned data assumptions and simplifications	☒	☐	9
**Outcomes and prioritization**	13	List and define all outcomes for which data will be sought, including prioritization of main and additional outcomes, with rationale	☒	☐	9,10
**Risk of bias in individual studies**	14	Describe anticipated methods for assessing risk of bias of individual studies, including whether this will be done at the outcome or study level, or both; state how this information will be used in data synthesis	☒	☐	10 (quantitative),12 (qualitative)
DATA					
**Synthesis**	15a	Describe criteria under which study data will be quantitatively synthesized	☒	☐	11-12
	15b	If data are appropriate for quantitative synthesis, describe planned summary measures, methods of handling data, and methods of combining data from studies, including any planned exploration of consistency (e.g., I^2, Kendall's tau)	☒	☐	11-12
	15c	Describe any proposed additional analyses (e.g., sensitivity or subgroup analyses, meta-regression)	☒	☐	12
	15d	If quantitative synthesis is not appropriate, describe the type of summary planned	☒	☐	12, 13
**Meta-bias(es)**	16	Specify any planned assessment of meta-bias(es) (e.g., publication bias across studies, selective reporting within studies)	☒	☐	10, 11

Section/topic	#	Checklist item	Information reported		Page number(s)
			Yes	No	
**Confidence in cumulative evidence**	17	Describe how the strength of the body of evidence will be assessed (e.g., GRADE)	☒	☐	13,14
