# Supplementary material for: Protocol for the SAFEST review: the Shock-Absorbing Flooring Effectiveness SysTematic review including older adults and staff in hospitals and care homes
Source: BMJ Open. 2020 Feb 17;10(2):e032315. doi: 10.1136/bmjopen-2019-032315 (PMC7044972; doi:10.1136/bmjopen-2019-032315)
Supplement: Supplementary data [file bmjopen-2019-032315supp002.pdf]

Medline search strategy

Search line	Terms
1	MH "Wounds and Injuries+
2	MH "Accidental Falls/PC”
3	MH "Hip Fractures+/PC"
4	falls
5	faller$
6	S1 OR S2 OR S3 OR S4 OR S5
7	MH "Aged+"
8	MH "Middle Aged"
9	Older
10	Senior$
11	elderly
12	S7 OR S8 OR S9 OR S10 OR S11
13	S6 AND S12
14	MH "Residential Facilities+"
15	MH "Long-Term Care"
16	MH "Institutionalization"
17	MH "Hospitalization"
18	MH "Subacute Care"
19	MH "Hospitals+"
20	MH "Hospital Units"
21	MH "Rehabilitation Centers"
22	MH "Inpatients"
23	MH "Geriatric Assessment"
24	("long stay" or "long term" or "acute" or "sub-acute" or "subacute" or "residential" or "hospital") N3 (care or ward# or hospital)
25	(rehabilitation or geriatric) N1 (ward# or hospital# or unit# or department#)
26	hostel$ or nursing home$
27	inpatient
28	resident$
29	institution$
30	S14 OR S15 OR S16 OR S17 OR S18 OR S19 OR S20 OR S21 OR S22 OR S23 OR S24 OR S25 OR S26 OR S27 OR S28 OR S29
31	S13 and S30
32	floor* NOT (pelvic floor OR sinus OR mouth)
33	carpet*
34	ground surface$
35	smartcell*
36	tarkett
37	softile
38	sorbashock
39	forbo
40	kradal
41	noraplan
42	MH "Floors and Floorcoverings"
43	S32 OR S33 OR S34 OR S35 OR S36 OR S37 OR S38 OR S39 OR S40 OR S41 OR S42
44	S31 AND S43
45	MH "Animals+"
46	MH "Humans"
47	S45 NOT S46
48	S44 NOT S47
49	S44 NOT S47<br>Limiters - Date of Publication: 20160501-
