# Supplementary material for: Protocol for the SAFEST review: the Shock-Absorbing Flooring Effectiveness SysTematic review including older adults and staff in hospitals and care homes
Source: BMJ Open. 2020 Feb 17;10(2):e032315. doi: 10.1136/bmjopen-2019-032315 (PMC7044972; doi:10.1136/bmjopen-2019-032315)
Supplement: Author's manuscript [file bmjopen-2019-032315.draft_revisions.pdf]

BMJ Open

BMJ Open is committed to open peer review. As part of this commitment we make the peer review history of every article we publish publicly available.

When an article is published we post the peer reviewers' comments and the authors' responses online. We also post the versions of the paper that were used during peer review. These are the versions that the peer review comments apply to.

The versions of the paper that follow are the versions that were submitted during the peer review process. They are not the versions of record or the final published versions. They should not be cited or distributed as the published version of this manuscript.

BMJ Open is an open access journal and the full, final, typeset and author-corrected version of record of the manuscript is available on our site with no access controls, subscription charges or pay-per-view fees (http://bmjopen.bmj.com).

If you have any questions on BMJ Open's open peer review process please email info.bmjopen@bmj.com

BMJ Open

Protocol for The SAFEST Review: The Shock-Absorbing Flooring Effectiveness SysTematic Review including older adults and staff in care settings

Journal:	*BMJ Open*
Manuscript ID	bmjopen-2019-032315
Article Type:	Protocol
Date Submitted by the Author:	12-Jun-2019
Complete List of Authors:	Drahota, Amy; University of Portsmouth, School of Health Sciences & Social Work<br>Felix, Lambert; University of Portsmouth, School of Health Sciences & Social Work<br>Keenan, Bethany; Cardiff University, School of Engineering<br>Lachance, Chantelle; University of Portsmouth, School of Health Sciences & Social Work<br>Laing , Andrew Laing ; University of Waterloo<br>Mackey, Dawn ; Simon Fraser University<br>Raftery, James; University of Southampton, Wessex Institute
Keywords:	Floors and Floorcoverings, Accidental Falls, Wounds and Injuries, Hospitals, Residential Facilities

SCHOLARONE™
Manuscripts

I, the Submitting Author has the right to grant and does grant on behalf of all authors of the Work (as defined in the below author licence), an exclusive licence and/or a non-exclusive licence for contributions from authors who are: i) UK Crown employees; ii) where BMJ has agreed a CC-BY licence shall apply, and/or iii) in accordance with the terms applicable for US Federal Government officers or employees acting as part of their official duties; on a worldwide, perpetual, irrevocable, royalty-free basis to BMJ Publishing Group Ltd ("BMJ") its licensees and where the relevant Journal is co-owned by BMJ to the co-owners of the Journal, to publish the Work in this journal and any other BMJ products and to exploit all rights, as set out in our licence.

The Submitting Author accepts and understands that any supply made under these terms is made by BMJ to the Submitting Author unless you are acting as an employee on behalf of your employer or a postgraduate student of an affiliated institution which is paying any applicable article publishing charge ("APC") for Open Access articles. Where the Submitting Author wishes to make the Work available on an Open Access basis (and intends to pay the relevant APC), the terms of reuse of such Open Access shall be governed by a Creative Commons licence – details of these licences and which Creative Commons licence will apply to this Work are set out in our licence referred to above.

Other than as permitted in any relevant BMJ Author's Self Archiving Policies, I confirm this Work has not been accepted for publication elsewhere, is not being considered for publication elsewhere and does not duplicate material already published. I confirm all authors consent to publication of this Work and authorise the granting of this licence.

For peer review only

Protocol for The SAFEST Review: The Shock-Absorbing Flooring Effectiveness SysTematic Review including older adults and staff in care settings

PROSPERO registration number: CRD42019118834

Corresponding Author

Amy Drahota, http://orcid.org/0000-0001-9772-0220
Principal Research Fellow,
School of Health Sciences & Social Work,
University of Portsmouth,
James Watson West,
2 King Richard 1st Rd, Portsmouth, Hampshire,
PO1 2FR, UK
Telephone: +44 (0)23 92 84 4432
Email: amy.drahota@port.ac.uk

Co-authors

1. Lambert M Felix, http://orcid.org/0000-0001-6517-9089
   Email: lambert.felix@port.ac.uk
   School of Health Sciences & Social Work, University of Portsmouth, Portsmouth, UK

2. Bethany Keenan, https://orcid.org/0000-0001-7787-2892
   Email: KeenanB@cardiff.ac.uk
   School of Engineering, University of Cardiff, Cardiff, UK

3. Chantelle C Lachance, http://orcid.org/0000-0001-5755-5160
   Email: chantelle.lachance@port.ac.uk
   School of Health Sciences & Social Work, University of Portsmouth, Portsmouth, UK

4. Andrew C Laing, https://orcid.org/0000-0001-8128-011X
   Email: actlaing@uwaterloo.ca
   University of Waterloo, Canada

5. Dawn C Mackey, http://orcid.org/0000-0001-9854-1486
   Email: dmackey@sfu.ca
   Simon Fraser University, Canada

6. James Raftery,
   Email: J.P.Raftery@soton.ac.uk
   Wessex Institute, University of Southampton, Southampton, UK

Word count: 4000
Key words: Floors and Floorcoverings; Accidental Falls; Wounds and Injuries; Hospitals; Residential Facilities.

For peer review only - http://bmjopen.bmj.com/site/about/guidelines.xhtml

ABSTRACT

Introduction: Falls in hospitals and care homes are a major issue of international concern. Inpatient falls are the most commonly reported safety incident in the United Kingdom's National Health Service (NHS), costing the NHS £630 million a year. Injurious falls are particularly life-limiting and costly. There is a growing body of evidence on shock-absorbing flooring for fall-related injury prevention; however, no systematic review exists to inform practice.

Methods and analysis: We will systematically identify, appraise, and summarise studies investigating the clinical and cost-effectiveness, and experiences of shock-absorbing flooring in hospitals and care homes. Our search will build on an extensive search conducted by a scoping review (inception to May 2016). We will search electronic databases (May 2016 – present), trial registries, and grey literature. We will screen reference lists, conduct forward citation searches, and liaise with study researchers. We will evaluate the influence of floors on fall-related injuries, falls, and staff work-related injuries through randomised and non-randomised studies, consider economic and qualitative evidence, and implementation factors. We will consider risk of bias, assess heterogeneity, and explore potential effect modifiers via subgroup analyses and sensitivity analyses. Where appropriate we will combine studies through meta-analysis. We will use the GRADE approach to evaluate the quality of evidence and present the results using summary of findings tables, and adhere to the PRISMA reporting guidelines.

Ethics and dissemination: We will follow the ethical principles of systematic review conduct, by attending to publication ethics, transparency, and rigour. Our dissemination plan includes peer-reviewed publication, presentations, press release, stakeholder symposium, patient video, and targeted knowledge-to-action reports. This review will inform decision-making around falls management in care settings and identify important directions for future research.

Funding and registration: The systematic review is funded by the NIHR HTA Programme (Project ref 17/148/11), and registered in PROSPERO (CRD42019118834).

ARTICLE SUMMARY

Strengths and limitations of this study

- This will be a mixed methods systematic review including randomised and non-randomised studies, economic and qualitative evidence;

For peer review only - http://bmjopen.bmj.com/site/about/guidelines.xhtml

- Studies will be assessed using the updated Cochrane risk of bias tools for quantitative evidence, and Joanna Briggs Institute method for qualitative studies;
- The quality of the evidence will be summarised using the GRADE approach, with the strength of the review's findings limited to the quantity and internal validity of the included studies;
- Analyses will be at the study level, which limits the scope for exploring moderating factors related to patient-level characteristics on the effectiveness of flooring interventions.
- We will be guided by the Knowledge-to-Action Framework to facilitate the translation of the findings into practice.

For peer review only

For peer review only - http://bmjopen.bmj.com/site/about/guidelines.xhtml

INTRODUCTION

Older adults, individuals living with frailty, and individuals with multiple morbidities are at greater risk of falls and fall-related injuries (1, 2). Falls in healthcare settings are a global concern (3). In the United Kingdom (UK), inpatient falls are the most commonly reported safety incident with over 250,000 reported per year in the National Health Service (NHS) in England alone. Of these falls, about 30% result in injury, causing a significant burden for individuals, carers, and healthcare resources due to the costs of continued and additional care and litigation (2). It is estimated that falls in hospitals cost the NHS about £630 million per year (2), which will be higher still if falls in other care settings (e.g. care homes) are included (4), and the wider impacts to the health and social care system are considered (5-6). Falls have a complex aetiology of intrinsic and extrinsic risk factors and no single solution effectively prevents them (3). It is proposed that 25-30% of inpatient falls are preventable, and the prevention of severe falls is a priority (2). A Cochrane review on hip protectors for hip fractures revealed that compliance with this intervention was poor and was a barrier to their use (7). Unlike hip protectors, manipulating the physical environment of care settings is a promising intervention for reducing injurious falls as it requires no compliance from patients or staff, and can accommodate the fact that injuries occur not just to the hip (8). Moreover, both The National Institute for Health and Care Excellence (NICE) Guideline (9), and the National Hip Fracture Database annual report (10) have highlighted the pressing need to investigate environmental adaptations to prevent falls and injuries.

Shock-absorbing flooring systems can reduce the impact forces of falls by decreasing the stiffness of the ground surface (11). However, softer floors could have a negative impact on older individuals' gait, particularly if they have complex health needs, potentially leading to increased falls risk (11-15). The potential benefits and risks of shock-absorbing floors may vary depending on the type of patient utilising them. Further adverse effects of shock-absorbing floors may present in staff if greater effort is required to manoeuvre rolling equipment, potentially increasing the risk of injuries (16).

There has been no comprehensive systematic review focussing on flooring interventions in healthcare settings for fall-related injury prevention. A recent scoping review of flooring interventions involved a thorough search of the literature through May 2016; however, it did not involve a critical appraisal or systematic synthesis (17). A systematic review of studies identified in the scoping review (16,18-25) as well as more recent studies (26-31), will provide a more reliable basis for decision-making and identify the next steps for research.

For peer review only - http://bmjopen.bmj.com/site/about/guidelines.xhtml

This publication is an abridged version of the full protocol available on the Health Technology Assessment website (32), and is registered on PROSPERO (CRD42019118834) (33). Any important protocol amendments will be published via these sites. We have conformed to the Preferred Reporting Items for Systematic Review and Meta-Analysis Protocols (PRISMA-P) (34) in the writing of this protocol (supplementary file 1).

Aims and Objectives

We aim to systematically review the evidence on shock-absorbing flooring use in care settings (hospitals and care homes) for fall-related injury prevention in older adults. Specifically, we will:

1. Assess the potential benefits (fall-related injury prevention) and risks (falls; staff injuries) of different flooring systems in care settings.
2. Assess the extent to which these potential benefits and risks may be modified by different study/setting, intervention, and participant characteristics.
3. Critically appraise and summarise current evidence on the resource use, costs and cost-effectiveness of shock-absorbing flooring in care settings for older adults, compared with standard flooring.
4. Summarise findings on the implementation of flooring interventions in the included studies.
5. Summarise the views and experiences of shock-absorbing flooring use from patients'/residents', staff, and visitors' perspectives.
6. Identify gaps in existing evidence.

METHODS

Eligibility criteria

Population

Our main population of interest is older adults in care settings. We have no set cut-off criteria for age, as chronological age may not be a good indicator of frailty (35). Studies must focus on adult populations to be included. We will exclude studies focussing solely on children.

Setting

Studies must have been conducted in a care setting (defined below) including hospitals (acute, sub-acute), intermediate and long-term care settings (nursing and care homes). Studies

For peer review only - http://bmjopen.bmj.com/site/about/guidelines.xhtml

conducted in people’s own homes, or other settings (e.g. playgrounds, sporting venues) will be excluded.

Care settings will be broadly defined as (36):

- Care home environments (a facility that provides: communal living facilities for long-term care; overnight accommodation; nursing or personal care; for people with illness, disability or dependence).
- Hospital environments (a facility that provides: communal care where there is an expectation that this care is time limited; overnight accommodation; nursing and personal care for people with illness and disability).

Interventions

Interventions may include flooring systems which have been purposely designed to prevent fall-related injuries (e.g. SmartCells, Sorbashock, Kradal), thick vinyl (>5mm thick; e.g. sports floors, such as Tarkett Omnisports Excel), carpet with or without underlay, and other combination flooring systems (e.g. vinyl overlays with padded underlays, such as foam or rubber, or wooden subfloors). Alternative terminology for the intervention may include variations on the terms: compliant flooring, safety flooring, soft flooring, impact absorbing flooring, energy absorbing flooring, low-impact flooring, dual stiffness flooring, low stiffness flooring, absorptive surfaces, cushioned flooring, rubber flooring, acoustic flooring, and carpet.

We will exclude studies reporting exclusively on mats as they are not permanently affixed to the floor and do not provide universal coverage or protection. We will exclude studies in which flooring is one component of a package of interventions and the effects of the floor cannot be disentangled from concurrent interventions.

Comparator

Our main comparison group is standard or rigid flooring (e.g. concrete, ≤2mm vinyl/resilient flooring). We will include head-to-head comparisons of different types of shock-absorbing flooring systems where possible.

Outcomes

The reporting of specific outcomes does not form part of our eligibility criteria.

Study Design

We will include randomised, non-randomised, observational, economic, and qualitative studies. Whilst randomised trials of flooring are feasible, the nature and logistics of the

For peer review only - http://bmjopen.bmj.com/site/about/guidelines.xhtml

intervention make observational and opportunistic quasi-experimental designs more practical. We will utilise the tables of study design features presented in the Cochrane Handbook (37) to classify included studies by their component design features. The following study designs will be eligible:

- Individually or cluster randomised controlled trials;
- Quasi experimental studies where allocation is non-random;
- Interrupted times series;
- Controlled before and after studies;
- Cohort studies;
- Case-control studies;
- Partial and full economic evaluations, based on a single study or model;
- Qualitative studies to explore experiences, attitudes, and perceptions towards flooring interventions.

We will exclude simple before and after studies measuring quantitative outcomes, with no evaluation of time trends or concurrent control.

Information sources and search strategy

We will build on the search already conducted in a scoping review (17), which had a search cut-off of May 2016. We will further assess the eligibility of clinical (12, 16, 18-26, 38-46) and cost-effectiveness (11, 21, 26, 40-41, 47-63) records identified in the scoping review. We will conduct our search from May 2016 to present, and will not apply any language restrictions. We will undertake a comprehensive search, as listed in Table 1, including electronic databases, grey literature, hand searches, citation screening, and expert consultation.

SEARCH TYPE	INFORMATION SOURCES
**Electronic Databases**	AgeLine (EBSCO)<br>CINAHL Complete (EBSCO)<br>MEDLINE (EBSCO)<br>NHS Economic Evaluation Database (Centre for Reviews and Dissemination)<br>Scopus<br>Web of Science (Thomson Reuters)
**Grey literature search**	**Clinical trial registries**<br>WHO International Clinical Trials Registry Platform (ICTRP)<br>**Theses/dissertations**<br>ProQuest Theses and Dissertations<br>**Abstracts/conference proceedings**<br>Biennial Conference of the Australian and New Zealand Falls Prevention Society<br>Canadian Association on Gerontology Annual Scientific and Educational Meeting

For peer review only - http://bmjopen.bmj.com/site/about/guidelines.xhtml

	Gerontological Society of America's Annual Scientific Meeting<br>International Society for Posture and Gait Research World Congress<br>World Conference of Gerontechnology<br>World Congress of the International Association of Gerontology and Geriatrics<br>**Websites**<br>Agency for Healthcare Research and Quality (AHRQ)<br>Canadian Agency for Drugs and Technologies in Health (CADTH)<br>NHS Improvement<br>NICE Guidelines<br>Open Grey (opengrey.eu)<br>Parachute Canada<br>The National Institute for Occupational Safety and Health (NIOSH)<br>The International Network of Agencies for Health Technology Assessment<br>UK Health Technology Assessment<br>US Center for Health Design<br>WHO Health Evidence Database (HEN)
**Hand searching and citation screening**	**Reference lists**<br>References of included studies<br>Forward citation searching of included studies in Web of Science<br>**Journal**<br>Age and Ageing

Table 1: List of information sources

We have refined the search strategy of the scoping review to focus on identifying studies of clinical and cost-effectiveness, and qualitative experiences. We have developed our strategy for MEDLINE (supplementary file 2) based on our eligibility criteria, using a combination of keyword synonyms and controlled vocabulary terms (e.g., MeSH). We will adapt the MEDLINE search for other information sources.

Study Records

Data management

We will import the search records into EndNote™ online, and use Covidence (54) to support duplicate record identification, screening, data collection, and risk of bias assessment processes, identification and resolution of discrepancies, and producing a PRISMA flow diagram (55). We will undertake data analysis in RevMan (56), and create Summary of Findings Tables and Evidence Profiles using GRADE Pro (57-58).

For peer review only - http://bmjopen.bmj.com/site/about/guidelines.xhtml

Selection process

We will screen titles, abstracts, and full reports independently in duplicate using an eligibility checklist. We will assess all records included in the clinical and cost-effectiveness sections of the scoping review at the full report stage. From the results of the updated search, we will begin by screening titles, and those that look potentially relevant we will review in abstract form. We will then screen the full texts of records that appear definitely or possibly relevant. We will resolve discrepancies through a third independent arbitrator.

Data collection process

Our theoretical framework of potential effect modifiers (Figure 1) will underpin the data collection process. We will develop and pilot the data collection form with a data collection manual to ensure consistency. Two reviewers will independently undertake data collection and assessment of risk of bias.

Data collection will include the following key components of information:

- Study identification
- Time/duration and geographical place of conduct
- Participant characteristics
- Intervention(s)
- Control(s)
- Outcome data acquisition: Method of falls reporting; Classification system of injuries; Identification of fractures (confirmation of diagnosis/type of fractures included); Identification of adverse effects.
- Setting
- Study design
- Risk of bias
- Outcomes data
- Patient and public involvement in the research
- Follow-up questions for study authors.

Outcomes and prioritization

There is no core outcome set specifically for flooring interventions; however we have considered the common outcome data set for fall injury prevention trials in community-dwelling populations (59) and the international consensus statement for trials on hip protectors (60). Recognising the unique features of our review and through stakeholder engagement (61) and

For peer review only - http://bmjopen.bmj.com/site/about/guidelines.xhtml

discussion with our public involvement group, we have prioritised the following outcome measures:

Primary outcomes:

- Injurious falls rate per 1000 patient-bed days;
- Falls rate per 1000 patient-bed days;

Secondary outcomes:

- Fractures per 1000 patient-bed days;
- Hip fractures per 1000 patient-bed days
- Number of fallers
- Number of fallers with injuries (none, minor, moderate, severe, death)
- Number of adverse events (e.g. staff injuries as defined by study authors)
- Number of fractures
- Number of hip fractures
- Qualitative outcomes (e.g. staff, patients/residents, visitors attitudes, views, and experiences)
- Economic outcomes (to include assessments of quality-adjusted life years)
- Process outcomes (e.g. ease of, or problems with, flooring installation)

Risk of bias in individual studies

Risk of bias assessment will be undertaken using the updated Cochrane risk of bias tool (ROB 2.0) for randomised trials (62) and the ROBINS-I (Risk Of Bias In Non-randomised Studies of Interventions) tool (63) for other quantitative designs. We will assess the risk of bias at the level of study results. Review authors will not be blinded during risk of bias assessments; however, where they have been involved in co-authoring an included study, assessments will be undertaken by at least two other independent reviewers. Supporting information and justification for judgements (high; low; some concerns) will be recorded for each risk of bias domain. We will follow the guidance to derive overall summary risk of bias judgements for each outcome, which will be used to inform our sensitivity analyses and GRADE assessments (57).

Data analysis (quantitative studies)

Dealing with missing data

For peer review only - http://bmjopen.bmj.com/site/about/guidelines.xhtml

We will seek further information from study authors where required. If missing data are from participant/cluster dropouts, we will conduct analyses based on the available data and include an assessment of the problem as part of our risk of bias judgements.

Measures of treatment effect

We will report rates of injurious falls, falls, and fractures using incidence rate ratios and 95% confidence intervals (CI). We will use risk ratios (95% CI) to describe number of fallers, number of participants with fall-related injuries, and number of participants with fall-related fractures. Where available we will also report hazard ratios for falls including all falls from recurrent fallers. For non-randomised studies, we will record the unadjusted and adjusted estimates and note the factors adjusted for. Where multiple adjusted estimates are presented, we will extract the estimate highlighted as the primary model by the authors, or where this is unclear, take the model which has adjusted for the most covariates. Where rate ratios or risk ratios are not reported, we will calculate them where feasible (37).

Where studies present a break-down of the severity of injuries (as ordinal outcome data, e.g. none, mild, moderate, severe, death), we will present these descriptively, and if studies have used similar categorisation systems, using figures where feasible. We will report adverse events to staff as a risk or rate ratio (per 100 working staff-days) where possible, or as the number of events observed during the follow-up period, if no clear denominator is known.

Unit of analysis issues

To avoid the issue of double counting, we will link multiple associated publications together. When primary studies include multiple study arms, we will either combine the groups (if logical) or include only one pair-wise comparison (intervention versus control) in any one analysis. In the case of cluster randomised trials we will take clustering into account, and plan to adjust the estimates using an intra-cluster correlation coefficient (ICC) borrowed from another similar study if required (37).

Assessment of reporting bias

Where possible, we will produce funnel plots with different plotting symbols to identify subgroups. We will only test for funnel plot asymmetry if there are sufficient data (at least 10 studies to be combined), and will use visual inspection of the plots to interpret the findings.

Data synthesis

Should meta-analysis be viable, we will opt to combine studies using a random-effects model, assuming that intervention effects are likely to vary across studies (Figure 1). We will use

For peer review only - http://bmjopen.bmj.com/site/about/guidelines.xhtml
For peer review only

generic inverse variance data type to produce forest plots in RevMan (56). Where evidence exists from randomised and non-randomised studies, we will report the data separately, giving more emphasis to the findings from randomised trials. We will organise non-randomised studies according to whether data collection was prospective or retrospective, and if controls were concurrent or historical. If appropriate, we will combine the data from randomised and non-randomised studies to provide an overall summary effect estimate.

Assessment of heterogeneity

We will explore heterogeneity irrespective of whether we decide to pool studies in a meta-analysis. Heterogeneity will be assessed through a combination of visual inspection of the forest plots, along with consideration of tests for homogeneity (Chi^2 with statistical significance set at $P < 0.10$), and measures for inconsistency (I^2) and heterogeneity (tau^2).

Where feasible, we plan to undertake subgroup analysis based on:

- Study design (randomised, non-randomised);
- Study setting (hospital, care home);
- Acuity of care (acute, sub-acute, intermediate, long-term care); and
- Flooring type (novel shock-absorbing flooring, thick vinyl/vinyl & underlay, carpet, wooden subfloor)

Sensitivity analyses

We will undertake sensitivity analyses based on:

- Risk of bias (e.g. removing studies at high risk of bias on the ROB2.0 tool, or critical/serious risk of bias on the ROBINS-I tool);
- Choice of effect estimates (e.g. where multiple adjusted estimates are reported, the analysis will be run on the most optimistic and pessimistic scenarios); and
- Adjustment for clustering where an ICC has been borrowed from another study (e.g. we will assess the impact of opting for more or less conservative adjustments).

Synthesis of Qualitative Studies

We will use a meta-aggregative approach to synthesise data from qualitative studies (64). We will derive generalizable statements, in the form of recommendations that can be used to guide end-users (e.g. NHS Chief Executives, care home managers, estates/facilities managers, healthcare designers and builders, health and social care professionals, patients, residents, and carers). We will critically assess the studies using the Joanna Briggs Institute's critical

For peer review only - http://bmjopen.bmj.com/site/about/guidelines.xhtml

appraisal tool (64). We will follow the data collection process as above, and use QSR NVivo software for data analysis (65).

Synthesis of economic evidence

We will align our approach for the incorporation of costs data to an exemplar systematic review by Garrison and colleagues (66). One reviewer (LF) will extract all data from included economic evaluations, which will be checked by an expert reviewer (JR). We will design our data extraction form based on the format and guidelines used to produce structured abstracts of full economic evaluations for inclusion in the NHS Economic Evaluation Database. We will assess the methodological quality of included economic evaluations through the use of recognised checklists based on guidelines for economic submissions to the British Medical Journal (for economic evaluations based on a single study) (67), and for quality assessment in economic decision-analytic models (for model-based economic evaluations) (68). Data extraction will include study characteristics such as country, settings, aims, and methodological aspects related to economic evaluation, individual items within the respective checklists (67-68), and the economic variables. We will collect the following economic variables, if reported: costs of flooring (purchasing, installation, maintenance); costs of falls based on injury, such as hospital resources (e.g. increased length of stay, additional surgery needs), and post-discharge healthcare cost (e.g. hospital readmission, outpatient visits); utility measures such as quality of life, life years and quality adjusted life years; and summary measures such as incremental cost-effectiveness ratio (ICERs), net monetary benefits, and value of information (VoI).

We will classify economic evaluations by type (*Partial evaluations*: 'outcome description', 'cost description', 'cost-outcome description', 'efficacy or effectiveness evaluation', or 'cost-analysis'; *Full economic evaluations*: 'cost-effectiveness analysis', 'cost-utility analysis', or 'cost-benefit analysis') and as either an economic evaluation based on a single study or a model-based economic evaluation. Where necessary, we will seek additional information from study authors.

We will tabulate and summarise the results narratively in the text. We will adjust all costs to 2019 Pound Sterling values using Gross Domestic Product deflators, and use relevant exchange rates for international comparisons.

For peer review only - http://bmjopen.bmj.com/site/about/guidelines.xhtml

Confidence in Cumulative Evidence

Quantitative Evidence

We will assess the quality of evidence across the included studies at outcome level for each comparison using GRADE (57), and incorporate these assessments into Summary of Findings (SoF) Tables using the GRADEpro software (58). Our main comparison will be 'shock-absorbing flooring versus standard flooring', and we will include separate SoF tables for hospitals and care homes. Supplementary SoF tables will be developed for different types of shock-absorbing floors versus standard flooring, and for head-to-head comparisons of different shock-absorbing flooring interventions.

We will include the following outcomes: 1) injurious falls rate per 1000 patient-bed days; 2) falls rate per 1000 patient-bed days; 3) fractures per 1000 patient bed days; 4) hip fractures per 1000 patient bed days; 5) number of fallers; 6) number of fallers with injuries (none, minor, moderate, severe); and 7) number of adverse events related to staff injuries. We will create 'Evidence profile' and Summary of Findings' tables (57). The GRADE system provides a grade of the overall quality of the evidence for each outcome on one of four levels: high, moderate, low, very low.

Qualitative Evidence

We will follow the CERQual group's recommendations to assess the quality of qualitative evidence included in the review (69). We will assess each review finding based on methodological limitations, coherence, adequacy of data, and relevance (70). We will make an overall assessment of confidence for each review finding on one of the four levels: high, moderate, low, very low. We will create 'CERQual Evidence profile', and 'Summary of Qualitative Findings (SoQF)' tables.

ENGAGEMENT WITH STAKEHOLDERS

We will consult with key stakeholders and a range of potential knowledge users during our review (i.e. small group meetings, one-to-one discussions, videoconferences, teleconferences, and email). Our Advisory Board includes the following knowledge users: Falls in older people NICE Guideline Developer; Safety and Improvement Clinical Lead (Leeds Teaching Hospitals NHS Trust); director/chairman of the Health Estates and Facilities Management Association; chairman of the National Care Association; public members; and shock-absorbing flooring researchers from health sciences and engineering disciplines in the UK and Canada. Collectively, members of the board possess the relevant expertise and decision-making authority to critically evaluate and implement shock-absorbing flooring

For peer review only - http://bmjopen.bmj.com/site/about/guidelines.xhtml

systems in high-risk environments such as hospitals and long-term care in the UK, and utilise systematic review evidence to inform future research.

An interactive process of communication between researchers and the Advisory Board will be used throughout the review process. We will involve the Board in a number of important ways: (1) in providing input on the design and implementation of the review; (2) as members of the project team who attend project meetings and inform us of emerging primary research evidence; (3) in the interpretation of findings and identification of research gaps; and (4) in the packaging and dissemination of the review's findings in a form that is relevant, practical and easily interpreted by other decision-makers and knowledge users.

PATIENT AND PUBLIC INVOLVEMENT (PPI)

Three public members engaged actively in the preparation of our original funding proposal. The members informed our decisions related to the systematic review methodology, particularly prioritising outcomes, confirming settings, and development of the theoretical framework.

The public members will participate in five specific PPI meetings over the course of the project. Each meeting will include a brief training session to explain the stage of the review the project is at, and the processes and tasks involved. They will contribute to the conduct of the systematic review in the following ways: (1) Commenting on the clarity and comprehensiveness of the protocol; Providing an independent judgement as to the fairness, transparency, and consistency of (2) the risk of bias, and (3) GRADE judgements made by the project team; (4) Providing feedback on the clarity of information presented in the Summary of Findings Tables, as well as the order and presentation of comparisons and subgroups; and (5) Providing feedback on the clarity, comprehensiveness, and presentation of the project outputs (including the Plain English Summary).

ETHICS AND DISSEMINATION

We do not need to obtain ethical review, as this is an evidence synthesis. Nonetheless, our ethical considerations (71) will relate to: (1) appropriateness of authorship on the final works; (2) avoidance of duplication in the publication of the findings; (3) avoiding plagiarism by ensuring that all reported findings are sufficiently cited and attributable to the source material; (4) transparency, in the form of acknowledging all contributions and competing interests ; (5) having due rigour in the data collection and reporting phases of the review to ensure the

For peer review only - http://bmjopen.bmj.com/site/about/guidelines.xhtml

accuracy of the findings; and (6) flagging suspected fraudulent or plagiarised research to the publishing journals.

Our research approach is underpinned by the Knowledge to Action Framework (72), and will ensure involvement of knowledge users with researchers throughout the process.
We will disseminate our research outputs using the following media:

- Open access peer-reviewed journal publication;
- Presentations at national and international conferences, and a webinar;
- Press release/social media with an item in relevant media outlets (e.g. The Conversation; The Health Estates and Facilities Management Association 'HEFMA Pulse' magazine);
- A half-day stakeholder symposium, the outputs of which will be made available online;
- A short video distilling the review findings via patient stories;
- Knowledge-To-Action Reports tailored to NHS Chief Executives, care home managers, and estates/facilities managers, healthcare designers and builders.

REFERENCES

1. Dhargave P, Sendhilkumar R. Prevalence of Risk Factors For falls among Elderly People Living in Long-Term Care Homes. *Journal of Clinical Gerontology and Geriatrics*. 2016;7(3):99-103.doi:10.1016/j.jcgg.2016.03.004
2. NHS Improvement. The Incidence and Costs of Inpatient Falls in Hospitals.2017. Available from: https://improvement.nhs.uk/documents/1471/Falls_report_July2017.v2.pdf. (accessed 27/03/19)
3. World Health Organization. Who Global Report on Falls Prevention in Older Age.2008. Available from: http://www.who.int/iris/handle/10665/43811. (accessed 14/03/19)
4. Rapp K, Becker C, Cameron ID, et al. Epidemiology of falls in residential aged care: analysis of more than 70,000 falls from residents of Bavarian nursing homes. *J Am Med Dir Assoc*. 2012;13(2):187.e1-6.doi: 10.1016/j.jamda.2011.06.011
5. Suzuki T, Sonoda S, Misawa K, et al. Incidence and Consequence of Falls in Inpatient Rehabilitation of Stroke Patients. *Experimental Aging Research*. 2005;31(4):457-69.doi:10.1080/03610730500206881
6. Vergara I, Vrotsou K, Orive M, et al. Factors Related to Functional Prognosis in Elderly Patients after Accidental Hip Fractures: A Prospective Cohort Study. *BMC Geriatr*. 2014;14:124.doi:10.1186/1471-2318-14-124

For peer review only - http://bmjopen.bmj.com/site/about/guidelines.xhtml

7. Santesso N, Carrasco-Labra A, Brignardello-Petersen R. Hip Protectors for Preventing Hip Fractures in Older People. *Cochrane Database Syst Rev*. 2014(3).doi:10.1002/14651858.CD001255.pub5

8. Fischer I, Krauss M, Dunagan W, et al. Patterns and Predictors of Inpatient Falls and Fall-Related Injuries in a Large Academic Hospital. *Infect Control Hosp Epidemiol*, 2005;26(10):822-827.doi:10.1086/502500

9. National Institute for Health and Care Excellence. Falls in Older People: Assessing Risk and Prevention. Clinical Guideline CG161.2013. Available from: https://www.nice.org.uk/guidance/cg161. (accessed 14/03/19)

10. Royal College of Physicians. National Hip Fracture Database Annual Report 2017.2017. Available from: https://www.nhfd.co.uk/2017report. (accessed 14/03/19)

11. Laing AC, Robinovitch SN. Low Stiffness Floors Can Attenuate Fall-Related Femoral Impact Forces by up to 50% without Substantially Impairing Balance in Older Women. *Accid Anal Prev*. 2009;41(3):642-50.doi:10.1016/j.aap.2009.03.001

12. Redfern MS, Moore PL, Yarsky CM. The Influence of Flooring on Standing Balance among Older Persons. *Hum Factors*. 1997;39(3):445-55.doi:10.1518/001872097778827043

13. Glinka MN, Cheema KP, Robinovitch SN, et al. Quantification of the Trade-Off between Force Attenuation and Balance Impairment in the Design of Compliant Safety Floors. *J Appl Biomech*. 2013;29(5):563-72.doi:10.1123/jab.29.5.563

14. Beach R, Jackson K, Bigelow KE, editors. Effect of Compliant Flooring on Postural Stability in an Older Adult Population. Proceedings of the 37th Annual Conference of the American Society of Biomechanics; 2013; Omaha, Nebraska, USA.

15. Stephens JM, Goldie PA. Walking Speed on Parquetry and Carpet after Stroke: Effect of Surface and Retest Reliability. *Clin Rehabil*. 1999;13(2):171-81.doi:10.1191/026921599668553798

16. Drahota AK, Ward D, Udell JE, et al. Pilot Cluster Randomised Controlled Trial of Flooring to Reduce Injuries from Falls in Wards for Older People. *Age Ageing*. 2013;42(5):633-40.doi:10.1093/ageing/aft067

17. Lachance CC, Jurkowski MP, Dymarz AC, et al. Compliant Flooring to Prevent Fall-Related Injuries in Older Adults: A Scoping Review of Biomechanical Efficacy, Clinical Effectiveness, Cost-Effectiveness, and Workplace Safety. *Plos One*. 2017;12(2):23.doi:10.1371/journal.pone.0171652

18. Donald IP, Pitt K, Armstrong E, et al. Preventing Falls on an Elderly Care Rehabilitation Ward. *Clin Rehabil*. 2000;14(2):178-85.doi:10.1191/026921500677888641

19. Gustavsson J, Bonander C, Andersson R, et al. Investigating the Fall-Injury Reducing Effect of Impact Absorbing Flooring among Female Nursing Home Residents: Initial Results. *Inj Prev*. 2015;21(5):320-4.doi:10.1136/injuryprev-2014-041468

For peer review only - http://bmjopen.bmj.com/site/about/guidelines.xhtml

20. Hanger HC, Hurley K, Hurring S, White A, editor Low Impact Flooring - Is It Practical in a Hospital? Proceedings of the 6th Biennial Australia and New Zealand Falls Prevention Conference; 2014; Sydney, Australia.

21. Healey F. Does Flooring Type Affect Risk of Injury in Older in-Patients? *Nurs Times*. 1994;90(27):40-1.https://www.ncbi.nlm.nih.gov/pubmed/8047456

22. Knoefel F, Patrick L, Taylor J, et al. Dual-Stiffness Flooring: Can It Reduce Fracture Rates Associated with Falls? *J Am Med Dir Assoc*. 2013;14(4):303-5.doi:10.1016/j.jamda.2012.12.077

23. Simpson AH, Lamb S, Roberts PJ, et al. Does the Type of Flooring Affect the Risk of Hip Fracture? *Age Ageing*. 2004;33(3):242-6.doi:10.1093/ageing/afh071

24. Warren CJ, Hanger HC. Fall and Fracture Rates Following a Change from Carpet to Vinyl Floor Coverings in a Geriatric Rehabilitation Hospital. A Longitudinal, Observational Study. *Clin Rehabil*. 2013;27(3):258-63.doi:10.1177/0269215512455530

25. Yarme J, Yarme H. Flooring and Safety. *Nursing Homes: Long Term Care Management*. 2001;50(10):82.

26. Lachance CC, Feldman F, Laing AC, et al. Study Protocol for the Flooring for Injury Prevention (Flip) Study: A Randomised Controlled Trial in Long-Term Care. *Inj. Prev.* 2016;22(6):453-60.doi:10.1136/injuryprev-2016-042008

27. Hanger HC. Low-Impact Flooring: Does It Reduce Fall-Related Injuries? *J Am Med Dir Assoc*. 2017;18(7):588-91.doi:10.1016/j.jamda.2017.01.012

28. Gustavsson J, Bonander C, Nilson F. A Quasi-Experimental Evaluation of Compliant Flooring in a Residential Care Setting. *Plos One*. 2018;13(7).doi:10.1371/journal.pone.0201290

29. Gustavsson J, Rahm G, Jernbro C, et al. Effects of Impact-Absorbing Flooring in Residential Care from the Perspectives of Enrolled Nurses. *Journal of Housing for the Elderly*. 2017;31(4):367-81.doi:10.1080/02763893.2017.1335670

30. Gustavsson J, Jernbro C, Nilson F. There Is More to Life Than Risk Avoidance - Elderly People's Experiences of Falls, Fall-Injuries and Compliant Flooring. *Int J Qual Stud Health Well-being*. 2018;13(1).doi:10.1080/17482631.2018.1479586

31. Lachance CC, Zaborska VO, Leung PM, et al. Perceptions About Compliant Flooring from Senior Managers in Lony-Terin Care. *Journal of Housing for the Elderly*. 2018;32(2):194-210.doi:10.1080/02763893.2018.1431584

32. Drahota A, Keenan B, Lachance C, et al. The SAFEST Review: The Shock-Absorbing Flooring Effectiveness SysTematic Review including older adults and staff in care settings. 17/148 HTA Researcher-led Evidence Synthesis. HTA - 17/148/11.2019. Available from: https://www.journalslibrary.nihr.ac.uk/programmes/hta/1714811/#/. (accessed 14/03/19)

For peer review only - http://bmjopen.bmj.com/site/about/guidelines.xhtml

33. Drahota A, Bell M, Farrell-Savage K, et al. The SAFEST Review: The Shock-Absorbing Flooring Effectiveness SysTematic Review including older adults and staff in care settings. PROSPERO 2019. CRD42019118834. Available from: http://www.crd.york.ac.uk/PROSPERO/display_record.php?ID=CRD42019118834. (accessed 14/03/19)

34. Moher D, Shamseer L, Clarke M, et al . *Preferred reporting items for systematic review and meta-analysis protocols (PRISMA-P)* 2015 *statement* . Syst Rev 2015;4:1.doi:10.1186/2046-4053-4-1

35. Mitnitski A, Howlett SE, Rockwood K. Heterogeneity of Human Aging and Its Assessment. *J Gerontol A Biol Sci Med Sci*. 2017;72(7):877-84.doi:10.1093/gerona/glw089

36. Ward D, Drahota A, Gal D, et al. Care Home Versus Hospital and Own Home Environments for Rehabilitation of Older People. *Cochrane Database Syst Rev*. 2008(4):CD003164.doi:10.1002/14651858.CD003164.pub2

37. Higgins JPT, Green S, editors. *Cochrane Handbook for Systematic Reviews of Interventions* Version 5.1.0 [Updated March 2011].Available from Http://Handbook.Cochrane.Org: The Cochrane Collaboration; 2011.

38. Birge SJ. Osteoporosis and Hip Fracture. *Clin Geriatr Med*. 1993;9(1):69-86. https://www.ncbi.nlm.nih.gov/pubmed/8443741

39. Brawley EC. Environment- a Silent Partner in Caregiving. In: Kaplan, M, Hoffman, SB, editors. Behaviors in Dementia: Best Practices for Successful Management: Health Professions Press; 1998. p. 107-24.

40. Canadian Agency for Drugs and Technologies in Health. Rubberized Flooring in Long Term Care: Clinical Effectiveness and Cost-Effectiveness. Rapid Response Report: Summary of Abstracts.2010. Available from: https://cadth.ca/sites/default/files/pdf/K0269_Rubber_Flooring_in_LTC_final.pdf. (accessed 14/03/19)

41. Center for Healthcare Enviornmental Management. Proper Flooring: A Critical Measure for Preventing Slips and Falls. *Healthc Hazard Manage Monit*. 2003;16(10):1-5, https://www.ncbi.nlm.nih.gov/pubmed/12817401

42. Cummings SR, Melton LJ. Epidemiology and Outcomes of Osteoporotic Fractures. *Lancet*. 2002;359(9319):1761-7.doi:10.1016/S0140-6736(02)08657-9

43. Hester AL. Preventing Injuries from Patient Falls. *American Nurse Today*. 2015;10(7):9-12.

44. Tibbitts GM. Patients Who Fall: How to Predict and Prevent Injuries. *Geriatrics*. 1996;51(9):24-8, 31. https://www.ncbi.nlm.nih.gov/pubmed/8814111

For peer review only

45. Tideiksaar R. Mechanisms of Falls in Community Residing Older Persons. *Pride Institute journal of long term home health care*. 1993.

46. Tideiksaar R, Fletcher B. Keeping Elderly on Their Feet. *Issues in Science and Technology*. 1989;5(3):78-81.

47. Hales M, Johnson JD, Asbury G, et al. Influence of Floor Covering Composition on Force Attenuation During Falls, Wheelchair Mobility, and Slip Resistance. *AATCC REV*. 2015;15:44–53.doi:10.14504/ar.15.6.4

48. Lange B. The Impact of Absorbent Floor in Reducing Hip Fractures: A Cost-Utility Analysis among Institutionalized Elderly: Karlstad University; 2012.

49. Latimer N, Dixon S, Drahota AK, et al. Cost--Utility Analysis of a Shock-Absorbing Floor Intervention to Prevent Injuries from Falls in Hospital Wards for Older People. *Age Ageing*. 2013;42(5):641-5.doi:10.1093/ageing/aft076

50. Njogu F, Brown P. Cost Effectiveness of Impact Absorbent Flooring in Reducing Fractures among Institutionalized Elderly.2008. Available from: https://mafiadoc.com/queue/cost-effectiveness-of-impact-absorbent-flooring-in-_5ba9567f097c47fb4e8b45e5.html.

51. Ryen L, Svensson M. Modelling the Cost-Effectiveness of Impact-Absorbing Flooring in Swedish Residential Care Facilities. *Eur J Public Health*. 2016;26(3):407-11.doi:10.1093/eurpub/ckv197.

52. Soangra R, Jones B, Lockhart TE, editors. Effects of Anti-Fatigue Flooring on Gait Parameters. Proceedings of the 54th Annual Meeting of the Human Factors and Ergonomics Society; 2010; San Francisco, California, USA: SAGE Publications.

53. Zacker C, Shea D. An Economic Evaluation of Energy-Absorbing Flooring to Prevent Hip Fractures. *Int J Technol Assess Health Care*. 1998;14(3):446-57, https://www.ncbi.nlm.nih.gov/pubmed/9780531

54. Covidence systematic review software. Veritas Health Innovation. Melborune, Australia: Available at www.covidence.org.

55. Moher D, Liberati A, Tetzlaff J, et al. Preferred Reporting Items for Systematic Reviews and Meta-Analyses: The Prisma Statement. *PLoS Med*. 2009;6(7):e1000097.doi:10.1371/journal.pmed.1000097

56. Review Manager (RevMan) [Computer program]. Version 5.3. Copenhagen: The Nordic Cochrane Centre, The *The Cochrane Collaboration*. 2014.

57. Schünemann H, Brożek J, Guyatt G, et al., editors. Grade Handbook for Grading Quality of Evidence and Strength of Recommendations. Updated October 2013.The GRADE Working Group, editor.Introduction to Grade Handbook. 2013.

58. GRADEpro GDT. Gradepro Guideline Development Tool [Software]. McMaster University, 2015 (developed by Evidence Prime, Inc.). Available from gradepro.org. 2015.

For peer review only - http://bmjopen.bmj.com/site/about/guidelines.xhtml

59. Lamb SE, Jørstad-Stein EC, Hauer K, et al. Development of a Common Outcome Data Set for Fall Injury Prevention Trials: The Prevention of Falls Network Europe Consensus. *J Am Geriatr Soc*. 2005;53(9):1618-22.doi:10.1111/j.1532-5415.2005.53455.x
60. Cameron ID, Robinovitch S, Birge S, et al. Hip Protectors: Recommendations for Conducting Clinical Trials--an International Consensus Statement (Part Ii). *Osteoporos Int*. 2010;21(1):1-10.doi:10.1007/s00198-009-1055-2
61. Lachance CC, Mackey DC. Feasibility of Compliant Flooring in Long-Term Care: Results from a Stakeholder Symposium. *Can J Aging*. 2018;37(1):84-94.doi:10.1017/S0714980817000551
62. Higgins JPT, Sterne JAC, Savović J, et al. A Revised Tool for Assessing Risk of Bias in Randomized Trials. In: Chandler J, McKenzie J, Boutron I, Welch V, editors. Cochrane Methods. *Cochrane Database of Systematic Reviews*. 10 (Suppl 1)2016.
63. Sterne JA, Hernán MA, Reeves BC, et al. Robins-I: A Tool for Assessing Risk of Bias in Non-Randomised Studies of Interventions. *BMJ*. 2016;355:i4919.doi:10.1136/bmj.i4919
64. Aromataris E, Munn Z, editors. Joanna Briggs Institute Reviewer's Manual.: The Joanna Briggs Institute. Available from https://reviewersmanual.joannabriggs.org/; 2017.
65. Soilemezi D, Drahota A, Crossland J, et al. The Role of the Home Environment in Dementia Care and Support: Systematic Review of Qualitative Research. *Dementia (London)*. 2017.doi:10.1177/1471301217692130
66. Garrison KR, Shemilt I, Donell S, et al. Bone Morphogenetic Protein (Bmp) for Fracture Healing in Adults. *Cochrane Database Syst Rev*. 2010(6):CD006950.doi:10.1002/14651858.CD006950.pub2
67. Drummond MF, Jefferson TO. Guidelines for Authors and Peer Reviewers of Economic Submissions to the BMJ. The Bmj Economic Evaluation Working Party. *BMJ*. 1996;313(7052):275-83,
68. Philips Z, Bojke L, Sculpher M, et al. Good Practice Guidelines for Decision-Analytic Modelling in Health Technology Assessment: A Review and Consolidation of Quality Assessment. *Pharmacoeconomics*. 2006;24(4):355-71.doi:10.2165/00019053-200624040-00006
69. The GRADE-CERQual Project Group. How to Use and Interpret Findings from Qualitative Evidence Syntheses Assessed Using the Cerqual Approach: A Practical Guide for Decision Makers in Health and Social Care and Other Users.2018. Available from: https://www.cerqual.org/. (accessed 13/03/19)
70. Lewin S, Booth A, Glenton C, et al. Applying Grade-Cerqual to Qualitative Evidence Synthesis Findings: Introduction to the Series. *Implement Sci*. 2018;13(Suppl 1):2.doi:10.1186/s13012-017-0688-3

For peer review only - http://bmjopen.bmj.com/site/about/guidelines.xhtml

71. Wager E, Wiffen PJ. Ethical Issues in Preparing and Publishing Systematic Reviews. *J Evid Based Med*. 2011;4(2):130-4.doi:10.1111/j.1756-5391.2011.01122.x

72. Graham ID, Logan J, Harrison MB, et al. Lost in Knowledge Translation: Time for a Map? *J Contin Educ Health Prof*. 2006;26(1):13-24.doi:10.1002/chp.47

AUTHOR STATEMENT

AD is Principal Investigator (guarantor) of the project, and was involved in the project conception and design, and drafting of the protocol. BK, CL, AL, and DM were all involved in the conception and design of the project, and revising the protocol content. JR contributed to the design of the health economics component and revising the protocol. LF contributed to the design of the Summary of Findings Tables component, and drafting of this manuscript. AD, BK, CL, and JR were co-applicants on the original funding proposal to HTA. All authors are also Advisory Board members and have read and approved the manuscript.

ACKNOWLEDGEMENTS

We would like to thank our public involvement members, Mrs Margaret Bell, Mrs Elizabeth Burden, and Mrs Joleen Tobias, and further members of our Advisory Board: Nadra Ahmed (National Care Association), Alison Cracknell (Leeds Teaching Hospitals NHS Trust), Kirsten Farrell-Savage (University of Portsmouth), Olanrewaju Okunribido (Health & Safety Executive), Jonathan Stewart (Health Estates and Facilities Management Association), Julie Windsor (NHS Improvement), and Anna Winfield (Leeds Teaching Hospitals NHS Trust). Our Advisory Board members have provided feedback and advice throughout the funding application process and when designing the protocol, and have approved the protocol. OO was also a co-applicant on the original funding proposal to HTA.

FUNDING STATEMENT

This report is independent research funded by the National Institute for Health Research (NIHR Health Technology Assessment, 17/148/11 – The SAFEST Review: The Shock-Absorbing Flooring Effectiveness SysTematic Review including older adults and staff in care settings). The views expressed in this publication are those of the authors and not necessarily those of the NHS, the National Institute for Health Research or the Department of Health and Social Care. The Health & Safety Executive are supporting 10 days of Olanrewaju Okunribido's time in kind on this project.

For peer review only - http://bmjopen.bmj.com/site/about/guidelines.xhtml

COMPETING INTERESTS STATEMENT

AD, CL, AL, and DM have undertaken studies that will be a part of this review.

AD and BK have been collaborating with members of the Health & Safety Laboratory (2018-present) on some unfunded academic research using a new testing procedure to assess the shock-absorbency of various floor coverings. Five flooring manufacturers delivered free samples to use in the project. AD and BK have no stake in any of these companies.

In 2015, AD was involved in a collaborative funding application with Polyflor for some SBRI Healthcare innovation funding. The application was short-listed but unsuccessful. AD has no stake in this company.

AL reports grants from SofSurfaces Inc, grants and personal fees from SorbaShock LLC, grants and personal fees from Viconic Sporting, outside the submitted work. AL is a member of an ASTM Work Group (WK38804) whose Technical Contact is the President of SATech. SATech has donated flooring materials to AL's laboratory that have formed the basis of several studies examining the biomechanical effectiveness of compliant flooring (i.e. safety flooring). He has never had (nor does he currently have) any financial links to the company.

CL is employed at the Canadian Agency for Drugs and Technologies in Health (CADTH).

JR is a member of the NIHR's HTA/EME editorial board (0.1 wte).

LF has nothing to disclose.

Additional Files

Supplementary file 1. PRISMA-P checklist.

Supplementary file 2. Medline search strategy

Figure 1. Theoretical framework of potential effect modifiers.

For peer review only

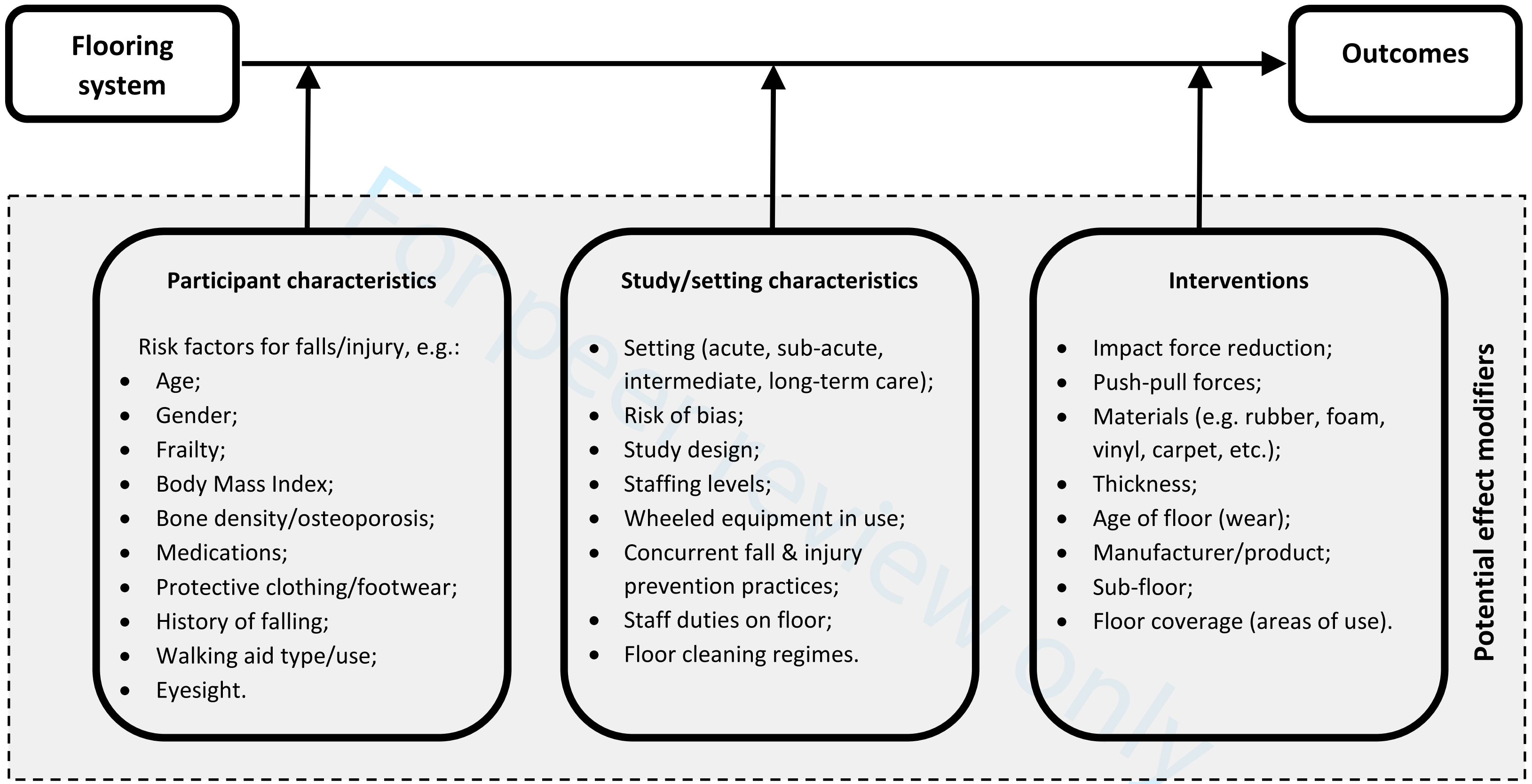
Flooring system
Outcomes
Participant characteristics
Risk factors for falls/injury, e.g.:
• Age;
• Gender;
• Frailty;
• Body Mass Index;
• Bone density/osteoporosis;
• Medications;
• Protective clothing/footwear;
• History of falling;
• Walking aid type/use;
• Eyesight.
Study/setting characteristics
• Setting (acute, sub-acute, intermediate, long-term care);
• Risk of bias;
• Study design;
• Staffing levels;
• Wheeled equipment in use;
• Concurrent fall & injury prevention practices;
• Staff duties on floor;
• Floor cleaning regimes.
Interventions
• Impact force reduction;
• Push-pull forces;
• Materials (e.g. rubber, foam, vinyl, carpet, etc.);
• Thickness;
• Age of floor (wear);
• Manufacturer/product;
• Sub-floor;
• Floor coverage (areas of use).
Potential effect modifiers

PRISMA- P 2015 Checklist

This checklist has been adapted from the operationalized checklist available from: http://www.prisma-statement.org/Extensions/Protocols.aspx which is based on Table 3 in Moher et al. Syst Rev. 2015;4(1):1. doi: 10.1186/2046-4053-4-1

Section/topic	#	Checklist item	Information reported		Page number(s)
			Yes	No	
**ADMINISTRATIVE INFORMATION**					
**Title**					
Identification	1a	Identify the report as a protocol of a systematic review	☒	☐	1
Update	1b	If the protocol is for an update of a previous systematic review, identify as such	☐	☒	n/a
**Registration**	2	If registered, provide the name of the registry (e.g., PROSPERO) and registration number in the Abstract	☒	☐	2
**Authors**					
Contact	3a	Provide name, institutional affiliation, and e-mail address of all protocol authors; provide physical mailing address of corresponding author	☒	☐	1
Contributions	3b	Describe contributions of protocol authors and identify the guarantor of the review	☒	☐	22
**Amendments**	4	If the protocol represents an amendment of a previously completed or published protocol, identify as such and list changes; otherwise, state plan for documenting important protocol amendments	☒	☐	5
**Support**					
Sources	5a	Indicate sources of financial or other support for the review	☒	☐	22
Sponsor	5b	Provide name for the review funder and/or sponsor	☒	☐	22
Role of sponsor/funder	5c	Describe roles of funder(s), sponsor(s), and/or institution(s), if any, in developing the protocol	☒	☐	22
**INTRODUCTION**					
**Rationale**	6	Describe the rationale for the review in the context of what is already known	☒	☐	4
**Objectives**	7	Provide an explicit statement of the question(s) the review will address with reference to participants, interventions, comparators, and outcomes (PICO)	☒	☐	5
**METHODS**					

For peer review only

Section/topic	#	Checklist item	Information reported Yes	Information reported No	Page number(s)
**Eligibility criteria**	8	Specify the study characteristics (e.g., PICO, study design, setting, time frame) and report characteristics (e.g., years considered, language, publication status) to be used as criteria for eligibility for the review	☒	☐	5-7
**Information sources**	9	Describe all intended information sources (e.g., electronic databases, contact with study authors, trial registers, or other grey literature sources) with planned dates of coverage	☒	☐	7-8
**Search strategy**	10	Present draft of search strategy to be used for at least one electronic database, including planned limits, such that it could be repeated	☒	☐	Supplementary file 2
STUDY RECORDS					
Data management	11a	Describe the mechanism(s) that will be used to manage records and data throughout the review	☒	☐	8
Selection process	11b	State the process that will be used for selecting studies (e.g., two independent reviewers) through each phase of the review (i.e., screening, eligibility, and inclusion in meta-analysis)	☒	☐	9
Data collection process	11c	Describe planned method of extracting data from reports (e.g., piloting forms, done independently, in duplicate), any processes for obtaining and confirming data from investigators	☒	☐	9,11
**Data items**	12	List and define all variables for which data will be sought (e.g., PICO items, funding sources), any pre-planned data assumptions and simplifications	☒	☐	9
**Outcomes and prioritization**	13	List and define all outcomes for which data will be sought, including prioritization of main and additional outcomes, with rationale	☒	☐	9,10
**Risk of bias in individual studies**	14	Describe anticipated methods for assessing risk of bias of individual studies, including whether this will be done at the outcome or study level, or both; state how this information will be used in data synthesis	☒	☐	10 (quantitative),12 (qualitative)
DATA					
**Synthesis**	15a	Describe criteria under which study data will be quantitatively synthesized	☒	☐	11-12
	15b	If data are appropriate for quantitative synthesis, describe planned summary measures, methods of handling data, and methods of combining data from studies, including any planned exploration of consistency (e.g., I^2, Kendall's tau)	☒	☐	11-12
	15c	Describe any proposed additional analyses (e.g., sensitivity or subgroup analyses, meta-regression)	☒	☐	12
	15d	If quantitative synthesis is not appropriate, describe the type of summary planned	☒	☐	12, 13
**Meta-bias(es)**	16	Specify any planned assessment of meta-bias(es) (e.g., publication bias across studies, selective reporting within studies)	☒	☐	10, 11

For peer review only

Section/topic	#	Checklist item	Information reported		Page number(s)
			Yes	No	
**Confidence in cumulative evidence**	17	Describe how the strength of the body of evidence will be assessed (e.g., GRADE)	☒	☐	13,14

For peer review only

Medline search strategy

Search line	Terms
1	MH "Wounds and Injuries+
2	MH "Accidental Falls/PC”
3	MH "Hip Fractures+/PC"
4	falls
5	faller$
6	S1 OR S2 OR S3 OR S4 OR S5
7	MH "Aged+"
8	MH "Middle Aged"
9	Older
10	Senior$
11	elderly
12	S7 OR S8 OR S9 OR S10 OR S11
13	S6 AND S12
14	MH "Residential Facilities+"
15	MH "Long-Term Care"
16	MH "Institutionalization"
17	MH "Hospitalization"
18	MH "Subacute Care"
19	MH "Hospitals+"
20	MH "Hospital Units"
21	MH "Rehabilitation Centers"
22	MH "Inpatients"
23	MH "Geriatric Assessment"
24	("long stay" or "long term" or "acute" or "sub-acute" or "subacute" or "residential" or "hospital") N3 (care or ward# or hospital)
25	(rehabilitation or geriatric) N1 (ward# or hospital# or unit# or department#)
26	hostel$ or nursing home$
27	inpatient
28	resident$
29	institution$
30	S14 OR S15 OR S16 OR S17 OR S18 OR S19 OR S20 OR S21 OR S22 OR S23 OR S24 OR S25 OR S26 OR S27 OR S28 OR S29
31	S13 and S30
32	floor* NOT (pelvic floor OR sinus OR mouth)
33	carpet*
34	ground surface$
35	smartcell*
36	tarkett
37	softile
38	sorbashock
39	forbo
40	kradal
41	noraplan
42	MH "Floors and Floorcoverings"
43	S32 OR S33 OR S34 OR S35 OR S36 OR S37 OR S38 OR S39 OR S40 OR S41 OR S42
44	S31 AND S43
45	MH "Animals+"
46	MH "Humans"
47	S45 NOT S46
48	S44 NOT S47
49	S44 NOT S47<br>Limiters - Date of Publication: 20160501-

For peer review only

BMJ Open

Protocol for The SAFEST Review: The Shock-Absorbing Flooring Effectiveness SysTematic Review including older adults and staff in hospitals and care homes

Journal:	*BMJ Open*
Manuscript ID	bmjopen-2019-032315.R1
Article Type:	Protocol
Date Submitted by the Author:	07-Nov-2019
Complete List of Authors:	Drahota, Amy; University of Portsmouth, School of Health & Care Professions<br>Felix, Lambert; University of Portsmouth, School of Health & Care Professions<br>Keenan, Bethany; Cardiff University, School of Engineering<br>Lachance, Chantelle; University of Portsmouth, School of Health & Care Professions<br>Laing , Andrew Laing ; University of Waterloo, Department of Kinesiology<br>Mackey, Dawn ; Simon Fraser University, Department of Biomedical Physiology and Kinesiology<br>Raftery, James; University of Southampton, Wessex Institute
**Primary Subject Heading**:	Evidence based practice
Secondary Subject Heading:	Geriatric medicine, Nursing, Health economics, Health services research
Keywords:	Floors and Floorcoverings, Accidental Falls, Wounds and Injuries, Hospitals, Residential Facilities

SCHOLARONE™ Manuscripts

I, the Submitting Author has the right to grant and does grant on behalf of all authors of the Work (as defined in the below author licence), an exclusive licence and/or a non-exclusive licence for contributions from authors who are: i) UK Crown employees; ii) where BMJ has agreed a CC-BY licence shall apply, and/or iii) in accordance with the terms applicable for US Federal Government officers or employees acting as part of their official duties; on a worldwide, perpetual, irrevocable, royalty-free basis to BMJ Publishing Group Ltd ("BMJ") its licensees and where the relevant Journal is co-owned by BMJ to the co-owners of the Journal, to publish the Work in this journal and any other BMJ products and to exploit all rights, as set out in our licence.

The Submitting Author accepts and understands that any supply made under these terms is made by BMJ to the Submitting Author unless you are acting as an employee on behalf of your employer or a postgraduate student of an affiliated institution which is paying any applicable article publishing charge ("APC") for Open Access articles. Where the Submitting Author wishes to make the Work available on an Open Access basis (and intends to pay the relevant APC), the terms of reuse of such Open Access shall be governed by a Creative Commons licence – details of these licences and which Creative Commons licence will apply to this Work are set out in our licence referred to above.

Other than as permitted in any relevant BMJ Author's Self Archiving Policies, I confirm this Work has not been accepted for publication elsewhere, is not being considered for publication elsewhere and does not duplicate material already published. I confirm all authors consent to publication of this Work and authorise the granting of this licence.

For peer review only

Protocol for The SAFEST Review: The Shock-Absorbing Flooring Effectiveness SysTematic Review including older adults and staff in hospitals and care homes

PROSPERO registration number: CRD42019118834

Corresponding Author

Amy Drahota, http://orcid.org/0000-0001-9772-0220
Reader in Health & Social Care Evidence & Evaluation,
School of Health & Care Professions,
University of Portsmouth,
St Andrews Court,
St Michael's Road, Portsmouth, Hampshire,
PO1 2PR, UK
Telephone: +44 (0)23 92 84 4432
Email: amy.drahota@port.ac.uk

Co-authors

1. Lambert M Felix, http://orcid.org/0000-0001-6517-9089
   Email: lambert.felix@port.ac.uk
   School of Health & Care Professions, University of Portsmouth, Portsmouth, UK

2. Bethany Keenan, https://orcid.org/0000-0001-7787-2892
   Email: KeenanB@cardiff.ac.uk
   School of Engineering, University of Cardiff, Cardiff, UK

3. Chantelle C Lachance, http://orcid.org/0000-0001-5755-5160
   Email: chantelle.lachance@port.ac.uk
   School of Health & Care Professions, University of Portsmouth, Portsmouth, UK

4. Andrew C Laing, https://orcid.org/0000-0001-8128-011X
   Email: actlaing@uwaterloo.ca
   Department of Kinesiology, University of Waterloo, Waterloo, Canada

5. Dawn C Mackey, http://orcid.org/0000-0001-9854-1486
   Email: dmackey@sfu.ca
   Department of Biomedical Physiology and Kinesiology, Simon Fraser University, Burnaby, Canada

6. James Raftery,
   Email: J.P.Raftery@soton.ac.uk
   Wessex Institute, University of Southampton, Southampton, UK

Word count: 4026
Key words: Floors and Floorcoverings; Accidental Falls; Wounds and Injuries; Hospitals; Residential Facilities.

For peer review only - http://bmjopen.bmj.com/site/about/guidelines.xhtml

ABSTRACT

Introduction: Falls in hospitals and care homes are a major issue of international concern. Inpatient falls are the most commonly reported safety incident in the United Kingdom's National Health Service (NHS), costing the NHS £630 million a year. Injurious falls are particularly life-limiting and costly. There is a growing body of evidence on shock-absorbing flooring for fall-related injury prevention; however, no systematic review exists to inform practice.

Methods and analysis: We will systematically identify, appraise, and summarise studies investigating the clinical and cost-effectiveness, and experiences of shock-absorbing flooring in hospitals and care homes. Our search will build on an extensive search conducted by a scoping review (inception to May 2016). We will search electronic databases (AgeLine, CINAHL, MEDLINE, NHS Economic Evaluation Database, Scopus, and Web of Science; May 2016 – present), trial registries, and grey literature. We will conduct backward and forward citation searches of included studies, and liaise with study researchers. We will evaluate the influence of floors on fall-related injuries, falls, and staff work-related injuries through randomised and non-randomised studies, consider economic and qualitative evidence, and implementation factors. We will consider risk of bias, assess heterogeneity, and explore potential effect modifiers via subgroup analyses and sensitivity analyses. Where appropriate we will combine studies through meta-analysis. We will use the GRADE approach to evaluate the quality of evidence and present the results using summary of findings tables, and adhere to the PRISMA reporting guidelines.

Ethics and dissemination: We will follow the ethical principles of systematic review conduct, by attending to publication ethics, transparency, and rigour. Our dissemination plan includes peer-reviewed publication, presentations, press release, stakeholder symposium, patient video, and targeted knowledge-to-action reports. This review will inform decision-making around falls management in care settings and identify important directions for future research.

Funding and registration: The systematic review is funded by the NIHR HTA Programme (Project ref 17/148/11), and registered in PROSPERO (CRD42019118834).

ARTICLE SUMMARY

Strengths and limitations of this study

- This will be a mixed methods systematic review including randomised and non-randomised clinical studies, economic and qualitative evidence;

For peer review only - http://bmjopen.bmj.com/site/about/guidelines.xhtml

- Studies will be assessed using the updated Cochrane risk of bias tools for quantitative evidence, and Joanna Briggs Institute method for qualitative studies;
- Analyses will be at the study level, which limits the scope for exploring moderating factors related to patient-level characteristics on the effectiveness of flooring interventions;
- The quality of the evidence will be summarised using the GRADE approach, with the strength of the review's findings limited to the quantity and internal validity of the included studies;
- We will be guided by the Knowledge-to-Action Framework to facilitate the translation of the findings into practice.

For peer review only

For peer review only - http://bmjopen.bmj.com/site/about/guidelines.xhtml

INTRODUCTION

Falls in health and social care settings are a major concern for older adults globally, causing morbidity, mortality and economic burden (1-3). Falls have been climbing the league tables of the leading causes of global disability-adjusted life years (4), with falls and injury rates in residential care settings substantially higher than that of older people living in the community (1). In the United Kingdom (UK), inpatient falls are the most commonly reported safety incident with over 250,000 reported per year in the National Health Service (NHS) in England alone (5). Falls have a complex aetiology of intrinsic (e.g. co-morbidities, cognitive function, mobility) and extrinsic (e.g. environmental design, staffing, footwear, medication) risk factors (3, 6-8) and no single solution effectively prevents them. A systematic review of falls prevention interventions in institutional settings (9), found low quality evidence, with uncertain conclusions for a range of interventions including: exercise, physiotherapy, sensor alarms, and multifactorial interventions. This review excluded studies targeting fall-related injury prevention, yet the prevention of severe falls is considered a priority (5). One of the most severe consequences of falls are hip fractures, but wearable hip protectors have poor compliance, which is a barrier to their use (10). Unlike hip protectors, manipulating the physical environment is a promising intervention for reducing injurious falls as it requires no compliance from patients or staff, and can accommodate other injury types.

Shock-absorbing flooring can reduce the impact forces of falls by decreasing the stiffness of the ground surface (11). However, softer floors could negatively impact on gait, potentially leading to increased falls risk (11-15). The potential benefits and risks of shock-absorbing floors may vary depending on the type of patient utilising them. Furthermore, adverse effects of shock-absorbing floors may present in staff if greater effort is required to manoeuvre rolling equipment, potentially increasing injury risk (16).

There has been no comprehensive systematic review focussing on flooring interventions in healthcare settings for fall-related injury prevention. A recent scoping review of flooring interventions involved a thorough search of the literature, however, it did not involve a critical appraisal or systematic synthesis (17). A systematic review of studies identified in the scoping review (16,18-25) as well as more recent studies (26-31), will provide a more reliable basis for decision-making and identify the next steps for research.

This publication is an abridged version of the full protocol (32), and is registered on PROSPERO (CRD42019118834) (33). Any important protocol amendments will be published on these platforms (32-33). We have conformed to the Preferred Reporting Items for

For peer review only - http://bmjopen.bmj.com/site/about/guidelines.xhtml

Systematic Review and Meta-Analysis Protocols (PRISMA-P) (34) in the writing of this protocol (supplementary file 1).

Aims and Objectives

We aim to systematically review the evidence on shock-absorbing flooring use in care settings (hospitals and care homes) for fall-related injury prevention in older adults. Specifically, we will:

1. Assess the potential benefits (fall-related injury prevention) and risks (falls; staff injuries) of different flooring systems in care settings.
2. Assess the extent to which these potential benefits and risks may be modified by different study/setting, intervention, and participant characteristics.
3. Critically appraise and summarise current evidence on the resource use, costs and cost-effectiveness of shock-absorbing flooring in care settings for older adults, compared with standard flooring.
4. Summarise findings on the implementation of flooring interventions in the included studies.
5. Summarise the views and experiences of shock-absorbing flooring use from staff, patients'/residents', and visitors' perspectives.
6. Identify gaps in existing evidence.

METHODS

Eligibility criteria

Population

The target population for the intervention to potentially benefit is older adults in care settings. We have no set cut-off criteria for age, as chronological age may not be a good indicator of frailty (35). Studies must focus on adult populations to be included; studies focussing solely on children will be excluded. We are also interested in staff outcomes.

Setting

Studies must have been conducted in a care setting (defined below) including hospitals (acute, sub-acute), intermediate and long-term care settings (nursing and care homes). Studies conducted in people's own homes, or other settings (e.g. playgrounds, sporting venues) will be excluded.

For peer review only - http://bmjopen.bmj.com/site/about/guidelines.xhtml

Care settings will be broadly defined as (36):

- Care home environments (a facility that provides: communal living facilities for long-term care; overnight accommodation; nursing or personal care; for people with illness, disability or dependence).
- Hospital environments (a facility that provides: communal care where there is an expectation that this care is time limited; overnight accommodation; nursing and personal care for people with illness and disability).

Interventions

Interventions may include flooring systems which have been purposely designed to prevent fall-related injuries (e.g. SmartCells, Sorbashock, Kradal), thick vinyl (>5mm thick; e.g. sports floors, such as Tarkett Omnisports Excel), carpet with or without underlay, and other combination flooring systems (e.g. vinyl overlays with padded underlays, such as foam or rubber, or wooden subfloors). Alternative terminology for the intervention may include variations on the terms: compliant flooring, safety flooring, soft flooring, impact absorbing flooring, energy absorbing flooring, low-impact flooring, dual stiffness flooring, low stiffness flooring, absorptive surfaces, cushioned flooring, rubber flooring, acoustic flooring, and carpet.

We will exclude studies reporting exclusively on mats as they are not permanently affixed to the floor and do not provide universal coverage or protection; mats have different implications for installation and practice and are not the focus of this review. Studies in which flooring is one component of a package of interventions and the effects of the floor cannot be distinguished from concurrent interventions will be excluded.

Comparator

Our main control group is standard or rigid flooring (e.g. concrete subfloor, ≤2mm vinyl/resilient flooring). We will include head-to-head comparisons of different types of shock-absorbing flooring systems where possible. Studies may compare any combination of flooring systems (subfloors and overlays).

Outcomes

The reporting of specific outcomes does not form part of our eligibility criteria for studies to be included in this review.

Study Design

We will include randomised, non-randomised, observational, economic, and qualitative studies. Whilst randomised trials of flooring are feasible, the nature and logistics of the

For peer review only - http://bmjopen.bmj.com/site/about/guidelines.xhtml

intervention make observational and opportunistic quasi-experimental designs more practical. Studies will be classified according to their component design features using the study design features presented in the Cochrane Handbook (37). The following study designs will be eligible:

- Individually or cluster randomised controlled trials;
- Quasi experimental studies where allocation is non-random;
- Interrupted times series;
- Controlled before and after studies;
- Cohort studies;
- Case-control studies;
- Partial and full economic evaluations, based on a single study or model;
- Qualitative studies to explore experiences, attitudes, and perceptions towards flooring interventions.

We will exclude simple before and after studies measuring quantitative outcomes, with no evaluation of time trends or concurrent control.

Information sources and search strategy

To avoid duplication of effort, we will build on the search already conducted in a scoping review (17), which completed its search in May 2016. The clinical (12, 16, 18-26, 38-46) and cost-effectiveness (11, 21, 26, 40-41, 47-63) records identified in the scoping review will be assessed for inclusion in the current review. We will continue the search from May 2016 to present, and will not apply any language restrictions. A comprehensive search, as listed in Table 1, will be undertaken, to include electronic databases, grey literature, hand searches, citation screening, and expert consultation.

SEARCH TYPE	INFORMATION SOURCES
**Electronic Databases**	AgeLine (EBSCO)<br>CINAHL Complete (EBSCO)<br>MEDLINE (EBSCO)<br>NHS Economic Evaluation Database (Centre for Reviews and Dissemination)<br>Scopus<br>Web of Science (Thomson Reuters)
**Grey literature search**	**Clinical trial registries**<br>WHO International Clinical Trials Registry Platform (ICTRP)<br>**Theses/dissertations**<br>ProQuest Theses and Dissertations<br>**Abstracts/conference proceedings**<br>Biennial Conference of the Australian and New Zealand Falls Prevention Society

For peer review only - http://bmjopen.bmj.com/site/about/guidelines.xhtml

	Canadian Association on Gerontology Annual Scientific and Educational Meeting<br>Gerontological Society of America's Annual Scientific Meeting<br>International Society for Posture and Gait Research World Congress<br>World Conference of Gerontechnology<br>World Congress of the International Association of Gerontology and Geriatrics<br>**Websites**<br>Agency for Healthcare Research and Quality (AHRQ)<br>Canadian Agency for Drugs and Technologies in Health (CADTH)<br>NHS Improvement<br>NICE Guidelines<br>Open Grey (opengrey.eu)<br>Parachute Canada<br>The National Institute for Occupational Safety and Health (NIOSH)<br>The International Network of Agencies for Health Technology Assessment<br>UK Health Technology Assessment<br>US Center for Health Design<br>WHO Health Evidence Database (HEN)
**Hand searching and citation screening**	**Reference lists**<br>References of included studies<br>Forward citation searching of included studies in Web of Science<br>**Journal**<br>Age and Ageing

Table 1: List of information sources

We have adapted the broader search strategy of the scoping review (17) to make it more specific to the current study (The SAFEST Review). The strategy for MEDLINE (supplementary file 2) is based on our eligibility criteria and uses a combination of keyword synonyms and controlled vocabulary terms (e.g., MeSH). We will adapt the MEDLINE search for other information sources.

Study Records

Data management

We will import the search records into EndNote^{TM} online and use Covidence (54) to support duplicate record identification, screening, data collection, and risk of bias assessment processes, identification and resolution of discrepancies, and producing a PRISMA flow diagram (55). Data analyses will be undertaken in RevMan (56), and Summary of Findings Tables and Evidence Profiles will be created using GRADE Pro (57-58).

For peer review only - http://bmjopen.bmj.com/site/about/guidelines.xhtml

Selection process

We will screen titles, abstracts, and full reports independently in duplicate using an eligibility checklist. All records included in the clinical and cost-effectiveness sections of the scoping review will be assessed at the full report stage. From the results of the updated search, we will begin by screening titles, and those that look potentially relevant will be reviewed in abstract form. We will then screen the full texts of records that appear definitely or possibly relevant. Discrepancies will be resolved through a third independent arbitrator.

Data collection process

Our theoretical framework of potential effect modifiers (Figure 1) will underpin the data collection process. We will develop and pilot the data collection form with a data collection manual. Two reviewers will independently undertake data collection and assessment of risk of bias.

Data collection will include the following key components of information:

- Study identification
- Time/duration and geographical place of conduct
- Participant characteristics
- Intervention(s)
- Control(s)
- Outcome data acquisition: Method of falls reporting; Classification system of injuries; Identification of fractures (confirmation of diagnosis/type of fractures included); Identification of adverse effects.
- Setting
- Study design
- Risk of bias
- Outcomes data
- Patient and public involvement in the research
- Follow-up questions for study authors.

Outcomes and prioritization

There is no core outcome set specifically for flooring interventions; however we have considered the common outcome data set for fall injury prevention trials in community-dwelling populations (59) and the international consensus statement for trials on hip protectors (60). Recognising the unique features of our review and through stakeholder engagement (61) and

For peer review only - http://bmjopen.bmj.com/site/about/guidelines.xhtml

discussion with our public involvement group, we have prioritised the following outcome measures:

- Primary outcomes:Injurious falls rate per 1000 patient-bed days;
- Falls rate per 1000 patient-bed days;

These measures assess the potential benefits and harms of flooring interventions for patients/residents, accounting for occupancy levels and follow-up time; injurious falls rate additionally accounts for variations to the underlying falls rate, as a pragmatic measure of effectiveness.

Secondary outcomes:

- Number of falls with injuries (e.g. none, minor, moderate, severe, death)
- Number of fractures
- Number of hip fractures
- Number of fallers (risk of falling ≥1 times)
- Number of adverse events (e.g. staff injuries as defined by study authors)
- Number of head injuries
- Fractures per 1000 patient-bed days;
- Hip fractures per 1000 patient-bed days
- Qualitative outcomes (e.g. staff, patients/residents, visitors attitudes, views, and experiences)
- Economic outcomes (to include assessments of quality-adjusted life years)
- Process outcomes (e.g. ease of, or problems with, flooring installation)

Risk of bias in individual studies

Risk of bias assessment will be undertaken using the updated Cochrane risk of bias tool (ROB 2.0) for randomised trials (62) and the ROBINS-I (Risk Of Bias In Non-randomised Studies of Interventions) tool (63) for other quantitative designs. We will assess the risk of bias at the level of study results. Review authors will not be blinded during risk of bias assessments; however, where they have been involved in co-authoring an included study, assessments will be undertaken by at least two other independent reviewers. Supporting information and justification for judgements (high; low; some concerns) will be recorded for each bias domain. We will follow the guidance to derive summary judgements for each outcome, which will be used to inform our sensitivity analyses and GRADE assessments (57).

For peer review only

Data analysis (quantitative studies)

Dealing with missing data

We will seek further information from study authors where required. If missing data are from participant/cluster dropouts, analyses will be based on the available data and an assessment of the problem will be included as part of our risk of bias judgements.

Measures of treatment effect

We will report rates of injurious falls, falls, and fractures using incidence rate ratios and 95% confidence intervals (CI). We will use risk ratios (95% CI) to describe number of fallers, number of falls with fall-related injuries, and number of participants with fall-related fractures or head injuries. Where available we will also report hazard ratios for falls including all falls from recurrent fallers. For non-randomised studies, we will record the unadjusted and adjusted estimates and note the factors adjusted for. Where multiple adjusted estimates are presented, we will extract the estimate highlighted as the primary model by the authors, or where this is unclear, take the model which has adjusted for the most covariates. Where rate ratios or risk ratios are not reported, we will calculate them where feasible (37).

Where studies present a break-down of the severity of injuries (as ordinal outcome data, e.g. none, mild, moderate, severe, death), we will present these descriptively, and if studies have used similar categorisation systems, using figures where feasible. We will report adverse events to staff as a risk or rate ratio (per 100 working staff-days) where possible, or as the number of events observed during the follow-up period, if no clear denominator is known.

Unit of analysis issues

To avoid the issue of double counting, we will link multiple associated publications together. When primary studies include multiple study arms, we will either combine the groups (if logical) or include only one pair-wise comparison (intervention versus control) in any one analysis. In the case of cluster randomised trials we will take clustering into account, and plan to adjust the estimates using an intra-cluster correlation coefficient (ICC) borrowed from another similar study if required (37).

Assessment of reporting bias

Where possible, we will produce funnel plots with different plotting symbols to identify subgroups. Funnel plot asymmetry will be tested if there are sufficient data (at least 10 studies to be combined), and visual inspection of the plots will be used to interpret the findings.

For peer review only - http://bmjopen.bmj.com/site/about/guidelines.xhtml

Data synthesis

Should meta-analysis be viable, studies will be combined using a random-effects model, assuming that intervention effects are likely to vary across studies (Figure 1). We will use the generic inverse variance data type to produce forest plots in RevMan (56); this method requires entering the natural logarithm of the rate ratio or risk ratio and its standard error for each study. We will use 95% CIs throughout. Where evidence exists from randomised and non-randomised studies, we will report the data separately, giving more emphasis to the findings from randomised trials. We will organise non-randomised studies according to whether data collection was prospective or retrospective, and if controls were concurrent or historical. If appropriate, we will combine the data from randomised and non-randomised studies to provide an overall summary effect estimate.

Assessment of heterogeneity

Heterogeneity will be explored irrespective of whether we decide to pool studies in a meta-analysis. Heterogeneity will be assessed through a combination of visual inspection of the forest plots, along with consideration of tests for homogeneity (Chi^2 with statistical significance set at $P < 0.10$), and measures for inconsistency (I^2) and heterogeneity (tau^2).

Where feasible, we plan to undertake subgroup analysis based on:

- Study design (randomised, non-randomised);
- Study setting (hospital, care home);
- Acuity of care (acute, sub-acute, intermediate, long-term care); and
- Flooring type (novel shock-absorbing flooring, thick vinyl/vinyl & underlay, carpet, wooden subfloor)

Sensitivity analyses

Sensitivity analyses will be undertaken based on:

- Risk of bias (e.g. removing studies at high risk of bias on the ROB2.0 tool, or critical/serious risk of bias on the ROBINS-I tool);
- Choice of effect estimates (e.g. where multiple adjusted estimates are reported, the analysis will be run on the most optimistic and pessimistic scenarios); and
- Adjustment for clustering where an ICC has been borrowed from another study (e.g. we will assess the impact of opting for more or less conservative adjustments).

For peer review only - http://bmjopen.bmj.com/site/about/guidelines.xhtml

Synthesis of Qualitative Studies

A meta-aggregative approach will be used to synthesise data from qualitative studies (64). We will derive generalizable statements, in the form of recommendations that can be used to guide end-users of the review (e.g. NHS Chief Executives, care home managers, estates/facilities managers, healthcare designers and builders, health and social care professionals, patients, residents, and carers). Studies will be critically assessed using the Joanna Briggs Institute's critical appraisal tool (64). We will follow the data collection process as above, and use QSR NVivo software for data analysis (65).

Synthesis of economic evidence

We will align our approach for the incorporation of costs data to an exemplar systematic review by Garrison and colleagues (66). One reviewer (LF) will extract all data from included economic evaluations, which will be checked by an expert reviewer (JR). Our data extraction form will be based on the format and guidelines used to produce structured abstracts of full economic evaluations for inclusion in the NHS Economic Evaluation Database. The methodological quality of included economic evaluations will be assessed through the use of recognised checklists based on guidelines for economic submissions to the British Medical Journal (for economic evaluations based on a single study) (67), and for quality assessment in economic decision-analytic models (for model-based economic evaluations) (68). Data extraction will include study characteristics such as country, settings, aims, and methodological aspects related to economic evaluation, individual items within the respective checklists (67-68), and the economic variables. We will collect the following economic variables, if reported: costs of flooring (purchasing, installation, maintenance); costs of falls based on injury, such as hospital resources (e.g. increased length of stay, additional surgery needs), and post-discharge healthcare cost (e.g. hospital readmission, outpatient visits); utility measures such as quality of life, life years and quality adjusted life years; and summary measures such as incremental cost-effectiveness ratio (ICERs), net monetary benefits, and value of information (VoI).

We will classify economic evaluations by type (*Partial evaluations*: 'outcome description', 'cost description', 'cost-outcome description', 'efficacy or effectiveness evaluation', or 'cost-analysis'; *Full economic evaluations*: 'cost-effectiveness analysis', 'cost-utility analysis', or 'cost-benefit analysis') and as either an economic evaluation based on a single study or a model-based economic evaluation. Where necessary, additional information from study authors will be sought.

For peer review only - http://bmjopen.bmj.com/site/about/guidelines.xhtml

Results will be tabulated and summarised narratively in the text. We will adjust all costs to 2019 Pound Sterling values using Gross Domestic Product deflators, and use relevant exchange rates for international comparisons.

Confidence in Cumulative Evidence

Quantitative Evidence

The quality of evidence will be assessed across the included studies at outcome level for each comparison using GRADE (57), and incorporated into Summary of Findings (SoF) Tables using the GRADEpro software (58). Our main comparison will be 'shock-absorbing flooring versus standard flooring', and we will include separate SoF tables for hospitals and care homes. Supplementary SoF tables will be developed for different types of shock-absorbing floors versus standard flooring, and for head-to-head comparisons of different shock-absorbing flooring interventions.

The following outcomes will be included: 1) injurious falls rate per 1000 patient-bed days; 2) falls rate per 1000 patient-bed days; 3) number of falls with injuries (e.g. none, minor, moderate, severe); 4) number of fractures; 5) number of hip fractures; 6) number of fallers; and 7) number of adverse events related to staff injuries. We will create supporting 'Evidence profile' tables (57). The GRADE system provides a grade of the overall quality of the evidence for each outcome on one of four levels: high, moderate, low, very low.

Qualitative Evidence

The CERQual group's recommendations will be followed to assess the quality of qualitative evidence included in the review (69). Each review finding will be assessed based on methodological limitations, coherence, adequacy of data, and relevance (70). We will make an overall assessment of confidence for each review finding on one of the four levels: high, moderate, low, very low. Assessments will be presented in 'CERQual Evidence profile', and 'Summary of Qualitative Findings (SoQF)' tables.

ENGAGEMENT WITH STAKEHOLDERS

We will consult with key stakeholders and a range of potential knowledge users during our review. Our Advisory Board includes the following knowledge users: Falls in older people NICE Guideline Developer; Safety and Improvement Clinical Lead (Leeds Teaching Hospitals NHS Trust); director/chairman of the Health Estates and Facilities Management Association; chairman of the National Care Association; public members; and shock-absorbing flooring

For peer review only - http://bmjopen.bmj.com/site/about/guidelines.xhtml

researchers from health sciences and engineering disciplines in the UK and Canada. Collectively, members of the board possess the relevant expertise and decision-making authority to critically evaluate and implement shock-absorbing flooring systems in high-risk environments such as hospitals and long-term care in the UK, and utilise systematic review evidence to inform future research.

An interactive process of communication between researchers and the Advisory Board will be used throughout the review process. We will involve the Board in a number of important ways: (1) in providing input on the design and implementation of the review; (2) as members of the project team who attend project meetings and inform us of emerging primary research evidence; (3) in the interpretation of findings and identification of research gaps; and (4) in the packaging and dissemination of the review's findings in a form that is relevant, practical and easily interpreted by other decision-makers and knowledge users.

PATIENT AND PUBLIC INVOLVEMENT (PPI)

Three public members engaged actively in the preparation of our funding proposal. They informed our decisions relating to methodology, particularly prioritising outcomes, confirming settings, and development of the theoretical framework.

The public members will participate in five specific PPI meetings over the course of the project. Each meeting will include a brief training session to explain the stage of the review the project is at, and the processes and tasks involved. They will contribute to the conduct of the systematic review in the following ways: (1) Commenting on the clarity and comprehensiveness of the protocol; Providing an independent judgement as to the fairness, transparency, and consistency of (2) the risk of bias, and (3) GRADE judgements made by the project team; (4) Providing feedback on the clarity of information presented in the Summary of Findings Tables, as well as the order and presentation of comparisons and subgroups; and (5) Providing feedback on the clarity, comprehensiveness, and presentation of the project outputs (including the Plain English Summary).

ETHICS AND DISSEMINATION

We do not need to obtain ethical review, as this is an evidence synthesis. Nonetheless, our ethical considerations (71) will relate to: 1) appropriateness of authorship on the final works; 2) avoidance of duplication in the publication of the findings; 3) avoiding plagiarism by ensuring that all reported findings are sufficiently cited and attributable to the source material; 4) transparency, in the form of acknowledging all contributions and competing interests ; 5)

For peer review only - http://bmjopen.bmj.com/site/about/guidelines.xhtml

having due rigour in the data collection and reporting phases of the review to ensure the accuracy of the findings; and 6) flagging suspected fraudulent or plagiarised research to the publishing journals.

Our research approach is underpinned by the Knowledge to Action Framework (72), and will ensure involvement of knowledge users with researchers throughout the process.
We will disseminate our research outputs using the following media:

- Open access peer-reviewed journal publication;
- Presentations at national and international conferences, and a webinar;
- Press release/social media with an item in relevant media outlets (e.g. The Conversation; The Health Estates and Facilities Management Association 'HEFMA Pulse' magazine);
- A half-day stakeholder symposium, the outputs of which will be made available online;
- A short video distilling the review findings via patient stories;
- Knowledge-To-Action Reports tailored to NHS Chief Executives, care home managers, and estates/facilities managers, healthcare designers and builders.

REFERENCES

1. Pointer S. Trends in hospitalised injury due to falls in older people, 2007–08 to 2016–17. Injury research and statistics series No 126. Canberra: Australian Institute of Health and Welfare 2019.

2. Turner N, Jones D, Dawson P, et al. The Perceptions and Rehabilitation Experience of Older People After Falling in the Hospital. *Rehabil Nurs*. 2019;44(3):141-150. doi: 10.1097/rnj.0000000000000107.

3. World Health Organization. WHO Global Report on Falls Prevention in Older Age. Geneva: World Health Organization. 2008. Available from: http://www.who.int/iris/handle/10665/43811. (accessed 14/03/19)

4. Kyu HH, Abate D, Abate KH, et al. Global, regional, and national disability-adjusted life-years (DALYs) for 359 diseases and injuries and healthy life expectancy (HALE) for 195 countries and territories, 1990–2017: a systematic analysis for the Global Burden of Disease Study 2017. *Lancet* 2018;392:1859–922.

5. NHS Improvement. The Incidence and Costs of Inpatient Falls in Hospitals. 2017. Available from: https://improvement.nhs.uk/documents/1471/Falls_report_July2017.v2.pdf. (accessed 22/10/19)

For peer review only - http://bmjopen.bmj.com/site/about/guidelines.xhtml

6. Dhargave P, Sendhilkumar R. Prevalence of Risk Factors For falls among Elderly People Living in Long-Term Care Homes. *Journal of Clinical Gerontology and Geriatrics*. 2016;7(3):99-103.doi:10.1016/j.jcgg.2016.03.004

7. Jørgensen TSH, Hansen AH, Sahlberg M, et al. Nationwide time trends and risk factors for in-hospital falls-related major injuries. *Int J Clin Pract*, 2015;69(6):703–709. doi: 10.1111/ijcp.12624

8. Najafpour Z, Godarzi Z, Arab M, et al. Risk Factors for Falls in Hospital In-Patients: A Prospective Nested Case Control Study. *Int J Health Policy Manag* 2019;8(5):300–306. doi: 10.15171/ijhpm.2019.11

9. Cameron ID, Dyer SM, Panagoda CE, et al. Interventions for preventing falls in older people in care facilities and hospitals. *Cochrane Database of Syst Rev.* 2018;9:Art. No.: CD005465. doi:10.1002/14651858.CD005465.pub4

10. Santesso N, Carrasco-Labra A, Brignardello-Petersen R. Hip Protectors for Preventing Hip Fractures in Older People. *Cochrane Database Syst Rev*. 2014;3.doi:10.1002/14651858.CD001255.pub5

11. Laing AC, Robinovitch SN. Low Stiffness Floors Can Attenuate Fall-Related Femoral Impact Forces by up to 50% without Substantially Impairing Balance in Older Women. *Accid Anal Prev*. 2009;41(3):642-50.doi:10.1016/j.aap.2009.03.001

12. Redfern MS, Moore PL, Yarsky CM. The Influence of Flooring on Standing Balance among Older Persons. *Hum Factors*. 1997;39(3):445-55.doi:10.1518/001872097778827043

13. Glinka MN, Cheema KP, Robinovitch SN, et al. Quantification of the Trade-Off between Force Attenuation and Balance Impairment in the Design of Compliant Safety Floors. *J Appl Biomech*. 2013;29(5):563-72.doi:10.1123/jab.29.5.563

14. Beach R, Jackson K, Bigelow KE, editors. Effect of Compliant Flooring on Postural Stability in an Older Adult Population. Proceedings of the 37th Annual Conference of the American Society of Biomechanics; 2013; Omaha, Nebraska, USA.

15. Stephens JM, Goldie PA. Walking Speed on Parquetry and Carpet after Stroke: Effect of Surface and Retest Reliability. *Clin Rehabil*. 1999;13(2):171-81.doi:10.1191/026921599668553798

16. Drahota AK, Ward D, Udell JE, et al. Pilot Cluster Randomised Controlled Trial of Flooring to Reduce Injuries from Falls in Wards for Older People. *Age Ageing*. 2013;42(5):633-40.doi:10.1093/ageing/aft067

17. Lachance CC, Jurkowski MP, Dymarz AC, et al. Compliant Flooring to Prevent Fall-Related Injuries in Older Adults: A Scoping Review of Biomechanical Efficacy, Clinical Effectiveness, Cost-Effectiveness, and Workplace Safety. *Plos One*. 2017;12(2):23.doi:10.1371/journal.pone.0171652

For peer review only - http://bmjopen.bmj.com/site/about/guidelines.xhtml

18. Donald IP, Pitt K, Armstrong E, et al. Preventing Falls on an Elderly Care Rehabilitation Ward. *Clin Rehabil*. 2000;14(2):178-85.doi:10.1191/026921500677888641

19. Gustavsson J, Bonander C, Andersson R, et al. Investigating the Fall-Injury Reducing Effect of Impact Absorbing Flooring among Female Nursing Home Residents: Initial Results. *Inj Prev*. 2015;21(5):320-4.doi:10.1136/injuryprev-2014-041468

20. Hanger HC, Hurley K, Hurring S, White A, editor Low Impact Flooring - Is It Practical in a Hospital? Proceedings of the 6th Biennial Australia and New Zealand Falls Prevention Conference; 2014; Sydney, Australia.

21. Healey F. Does Flooring Type Affect Risk of Injury in Older in-Patients? *Nurs Times*. 1994;90(27):40-1.https://www.ncbi.nlm.nih.gov/pubmed/8047456

22. Knoefel F, Patrick L, Taylor J, et al. Dual-Stiffness Flooring: Can It Reduce Fracture Rates Associated with Falls? *J Am Med Dir Assoc*. 2013;14(4):303-5.doi:10.1016/j.jamda.2012.12.077

23. Simpson AH, Lamb S, Roberts PJ, et al. Does the Type of Flooring Affect the Risk of Hip Fracture? *Age Ageing*. 2004;33(3):242-6.doi:10.1093/ageing/afh071

24. Warren CJ, Hanger HC. Fall and Fracture Rates Following a Change from Carpet to Vinyl Floor Coverings in a Geriatric Rehabilitation Hospital. A Longitudinal, Observational Study. *Clin Rehabil*. 2013;27(3):258-63.doi:10.1177/0269215512455530

25. Yarme J, Yarme H. Flooring and Safety. *Nursing Homes: Long Term Care Management*. 2001;50(10):82.

26. Lachance CC, Feldman F, Laing AC, et al. Study Protocol for the Flooring for Injury Prevention (Flip) Study: A Randomised Controlled Trial in Long-Term Care. *Inj. Prev.* 2016;22(6):453-60.doi:10.1136/injuryprev-2016-042008

27. Hanger HC. Low-Impact Flooring: Does It Reduce Fall-Related Injuries? *J Am Med Dir Assoc*. 2017;18(7):588-91.doi:10.1016/j.jamda.2017.01.012

28. Gustavsson J, Bonander C, Nilson F. A Quasi-Experimental Evaluation of Compliant Flooring in a Residential Care Setting. *Plos One*. 2018;13(7).doi:10.1371/journal.pone.0201290

29. Gustavsson J, Rahm G, Jernbro C, et al. Effects of Impact-Absorbing Flooring in Residential Care from the Perspectives of Enrolled Nurses. *Journal of Housing for the Elderly*. 2017;31(4):367-81.doi:10.1080/02763893.2017.1335670

30. Gustavsson J, Jernbro C, Nilson F. There Is More to Life Than Risk Avoidance - Elderly People's Experiences of Falls, Fall-Injuries and Compliant Flooring. *Int J Qual Stud Health Well-being*. 2018;13(1).doi:10.1080/17482631.2018.1479586

31. Lachance CC, Zaborska VO, Leung PM, et al. Perceptions About Compliant Flooring from Senior Managers in Lony-Terin Care. *Journal of Housing for the Elderly*. 2018;32(2):194-210.doi:10.1080/02763893.2018.1431584

For peer review only - http://bmjopen.bmj.com/site/about/guidelines.xhtml

32. Drahota A, Keenan B, Lachance C, et al. The SAFEST Review: The Shock-Absorbing Flooring Effectiveness SysTematic Review including older adults and staff in care settings. 17/148 HTA Researcher-led Evidence Synthesis. HTA - 17/148/11.2019. Available from: https://www.journalslibrary.nihr.ac.uk/programmes/hta/1714811/#/. (accessed 14/03/19)

33. Drahota A, Bell M, Farrell-Savage K, et al. The SAFEST Review: The Shock-Absorbing Flooring Effectiveness SysTematic Review including older adults and staff in care settings. PROSPERO 2019. CRD42019118834. Available from: http://www.crd.york.ac.uk/PROSPERO/display_record.php?ID=CRD42019118834. (accessed 14/03/19)

34. Moher D, Shamseer L, Clarke M, et al. *Preferred reporting items for systematic review and meta-analysis protocols (PRISMA-P) 2015 statement* . Syst Rev 2015;4:1.doi:10.1186/2046-4053-4-1

35. Mitnitski A, Howlett SE, Rockwood K. Heterogeneity of Human Aging and Its Assessment. *J Gerontol A Biol Sci Med Sci*. 2017;72(7):877-84.doi:10.1093/gerona/glw089

36. Ward D, Drahota A, Gal D, et al. Care Home Versus Hospital and Own Home Environments for Rehabilitation of Older People. *Cochrane Database Syst Rev*. 2008(4):CD003164.doi:10.1002/14651858.CD003164.pub2

37. Higgins JPT, Thomas J, Chandler J, et al., editors. *Cochrane Handbook for Systematic Reviews of Interventions* Version 6.0 (updated July 2019). Cochrane, 2019. Available from www.training.cochrane.org/handbook.

38. Birge SJ. Osteoporosis and Hip Fracture. *Clin Geriatr Med*. 1993;9(1):69-86. https://www.ncbi.nlm.nih.gov/pubmed/8443741

39. Brawley EC. Environment- a Silent Partner in Caregiving. In: Kaplan, M, Hoffman, SB, editors. Behaviors in Dementia: Best Practices for Successful Management: Health Professions Press; 1998. p. 107-24.

40. Canadian Agency for Drugs and Technologies in Health. Rubberized Flooring in Long Term Care: Clinical Effectiveness and Cost-Effectiveness. Rapid Response Report: Summary of Abstracts.2010. Available from: https://cadth.ca/sites/default/files/pdf/K0269_Rubber_Flooring_in_LTC_final.pdf. (accessed 14/03/19)

41. Center for Healthcare Enviornmental Management. Proper Flooring: A Critical Measure for Preventing Slips and Falls. *Healthc Hazard Manage Monit*. 2003;16(10):1-5, https://www.ncbi.nlm.nih.gov/pubmed/12817401

42. Cummings SR, Melton LJ. Epidemiology and Outcomes of Osteoporotic Fractures. *Lancet*. 2002;359(9319):1761-7.doi:10.1016/S0140-6736(02)08657-9

For peer review only - http://bmjopen.bmj.com/site/about/guidelines.xhtml

43. Hester AL. Preventing Injuries from Patient Falls. *American Nurse Today*. 2015;10(7):9-12.

44. Tibbitts GM. Patients Who Fall: How to Predict and Prevent Injuries. *Geriatrics*. 1996;51(9):24-8, 31. https://www.ncbi.nlm.nih.gov/pubmed/8814111

45. Tideiksaar R. Mechanisms of Falls in Community Residing Older Persons. *Pride Institute journal of long term home health care*. 1993.

46. Tideiksaar R, Fletcher B. Keeping Elderly on Their Feet. *Issues in Science and Technology*. 1989;5(3):78-81.

47. Hales M, Johnson JD, Asbury G, et al. Influence of Floor Covering Composition on Force Attenuation During Falls, Wheelchair Mobility, and Slip Resistance. *AATCC REV*. 2015;15:44–53.doi:10.14504/ar.15.6.4

48. Lange B. The Impact of Absorbent Floor in Reducing Hip Fractures: A Cost-Utility Analysis among Institutionalized Elderly: Karlstad University; 2012.

49. Latimer N, Dixon S, Drahota AK, et al. Cost--Utility Analysis of a Shock-Absorbing Floor Intervention to Prevent Injuries from Falls in Hospital Wards for Older People. *Age Ageing*. 2013;42(5):641-5.doi:10.1093/ageing/aft076

50. Njogu F, Brown P. Cost Effectiveness of Impact Absorbent Flooring in Reducing Fractures among Institutionalized Elderly.2008. Available from: https://mafiadoc.com/queue/cost-effectiveness-of-impact-absorbent-flooring-in-_5ba9567f097c47fb4e8b45e5.html.

51. Ryen L, Svensson M. Modelling the Cost-Effectiveness of Impact-Absorbing Flooring in Swedish Residential Care Facilities. *Eur J Public Health*. 2016;26(3):407-11.doi:10.1093/eurpub/ckv197.

52. Soangra R, Jones B, Lockhart TE, editors. Effects of Anti-Fatigue Flooring on Gait Parameters. Proceedings of the 54th Annual Meeting of the Human Factors and Ergonomics Society; 2010; San Francisco, California, USA: SAGE Publications.

53. Zacker C, Shea D. An Economic Evaluation of Energy-Absorbing Flooring to Prevent Hip Fractures. *Int J Technol Assess Health Care*. 1998;14(3):446-57, https://www.ncbi.nlm.nih.gov/pubmed/9780531

54. Covidence systematic review software. Veritas Health Innovation. Melborune, Australia: Available at www.covidence.org.

55. Moher D, Liberati A, Tetzlaff J, et al. Preferred Reporting Items for Systematic Reviews and Meta-Analyses: The Prisma Statement. *PLoS Med*. 2009;6(7):e1000097.doi:10.1371/journal.pmed.1000097

56. Review Manager (RevMan) [Computer program]. Version 5.3. Copenhagen: The Nordic Cochrane Centre, The *The Cochrane Collaboration*. 2014.

For peer review only

57. Schünemann H, Brożek J, Guyatt G, et al., editors. Grade Handbook for Grading Quality of Evidence and Strength of Recommendations. Updated October 2013.The GRADE Working Group, editor.Introduction to Grade Handbook. 2013.

58. GRADEpro GDT. Gradepro Guideline Development Tool [Software]. McMaster University, 2015 (developed by Evidence Prime, Inc.). Available from gradepro.org. 2015.

59. Lamb SE, Jørstad-Stein EC, Hauer K, et al. Development of a Common Outcome Data Set for Fall Injury Prevention Trials: The Prevention of Falls Network Europe Consensus. *J Am Geriatr Soc*. 2005;53(9):1618-22.doi:10.1111/j.1532-5415.2005.53455.x

60. Cameron ID, Robinovitch S, Birge S, et al. Hip Protectors: Recommendations for Conducting Clinical Trials--an International Consensus Statement (Part Ii). *Osteoporos Int*. 2010;21(1):1-10.doi:10.1007/s00198-009-1055-2

61. Lachance CC, Mackey DC. Feasibility of Compliant Flooring in Long-Term Care: Results from a Stakeholder Symposium. *Can J Aging*. 2018;37(1):84-94.doi:10.1017/S0714980817000551

62. Higgins JPT, Sterne JAC, Savović J, et al. A Revised Tool for Assessing Risk of Bias in Randomized Trials. In: Chandler J, McKenzie J, Boutron I, Welch V, editors. Cochrane Methods. *Cochrane Database of Systematic Reviews*. 10 (Suppl 1)2016.

63. Sterne JA, Hernán MA, Reeves BC, et al. Robins-I: A Tool for Assessing Risk of Bias in Non-Randomised Studies of Interventions. *BMJ*. 2016;355:i4919.doi:10.1136/bmj.i4919

64. Aromataris E, Munn Z, editors. Joanna Briggs Institute Reviewer's Manual.: The Joanna Briggs Institute. Available from https://reviewersmanual.joannabriggs.org/; 2017.

65. Soilemezi D, Drahota A, Crossland J, et al. The Role of the Home Environment in Dementia Care and Support: Systematic Review of Qualitative Research. *Dementia (London)*. 2017.doi:10.1177/1471301217692130

66. Garrison KR, Shemilt I, Donell S, et al. Bone Morphogenetic Protein (Bmp) for Fracture Healing in Adults. *Cochrane Database Syst Rev*. 2010(6):CD006950.doi:10.1002/14651858.CD006950.pub2

67. Drummond MF, Jefferson TO. Guidelines for Authors and Peer Reviewers of Economic Submissions to the BMJ. The Bmj Economic Evaluation Working Party. *BMJ*. 1996;313(7052):275-83,

68. Philips Z, Bojke L, Sculpher M, et al. Good Practice Guidelines for Decision-Analytic Modelling in Health Technology Assessment: A Review and Consolidation of Quality Assessment. *Pharmacoeconomics*. 2006;24(4):355-71.doi:10.2165/00019053-200624040-00006

69. The GRADE-CERQual Project Group. How to Use and Interpret Findings from Qualitative Evidence Syntheses Assessed Using the Cerqual Approach: A Practical Guide for

For peer review only - http://bmjopen.bmj.com/site/about/guidelines.xhtml

Decision Makers in Health and Social Care and Other Users.2018. Available from: https://www.cerqual.org/. (accessed 13/03/19)

70. Lewin S, Booth A, Glenton C, et al. Applying Grade-Cerqual to Qualitative Evidence Synthesis Findings: Introduction to the Series. *Implement Sci*. 2018;13(Suppl 1):2.doi:10.1186/s13012-017-0688-3

71. Wager E, Wiffen PJ. Ethical Issues in Preparing and Publishing Systematic Reviews. *J Evid Based Med*. 2011;4(2):130-4.doi:10.1111/j.1756-5391.2011.01122.x

72. Graham ID, Logan J, Harrison MB, et al. Lost in Knowledge Translation: Time for a Map? *J Contin Educ Health Prof*. 2006;26(1):13-24.doi:10.1002/chp.47

AUTHOR STATEMENT

AD is Principal Investigator (guarantor) of the project, and was involved in the project conception and design, and drafting of the protocol. BK, CL, AL, and DM were all involved in the conception and design of the project, and revising the protocol content. JR contributed to the design of the health economics component and revising the protocol. LF contributed to the design of the Summary of Findings Tables component, and drafting of this manuscript. AD, BK, CL, and JR were co-applicants on the original funding proposal to HTA. All authors are also Advisory Board members and have read and approved the manuscript.

ACKNOWLEDGEMENTS

We would like to thank our public involvement members, Mrs Margaret Bell, Mrs Elizabeth Burden, and Mrs Joleen Tobias, and further members of our Advisory Board: Nadra Ahmed (National Care Association), Alison Cracknell (Leeds Teaching Hospitals NHS Trust), Kirsten Farrell-Savage (University of Portsmouth), Olanrewaju Okunribido (Health & Safety Executive), Jonathan Stewart (Health Estates and Facilities Management Association), Julie Windsor (NHS Improvement), and Anna Winfield (Leeds Teaching Hospitals NHS Trust). Our Advisory Board members have provided feedback and advice throughout the funding application process and when designing the protocol, and have approved the protocol. OO was also a co-applicant on the original funding proposal to HTA.

FUNDING STATEMENT

This report is independent research funded by the National Institute for Health Research (NIHR Health Technology Assessment, 17/148/11 – The SAFEST Review: The Shock-Absorbing Flooring Effectiveness SysTematic Review including older adults and staff in care

For peer review only - http://bmjopen.bmj.com/site/about/guidelines.xhtml

settings). The views expressed in this publication are those of the authors and not necessarily those of the NHS, the National Institute for Health Research or the Department of Health and Social Care. The Health & Safety Executive are supporting 10 days of Olanrewaju Okunribido's time in kind on this project.

COMPETING INTERESTS STATEMENT

AD, CL, AL, and DM have undertaken studies that will be a part of this review.

AD and BK have been collaborating with members of the Health & Safety Laboratory (2018-present) on some unfunded academic research using a new testing procedure to assess the shock-absorbency of various floor coverings. Five flooring manufacturers delivered free samples to use in the project. AD and BK have no stake in any of these companies.

In 2015, AD was involved in a collaborative funding application with Polyflor for some SBRI Healthcare innovation funding. The application was short-listed but unsuccessful. AD has no stake in this company.

AL reports grants from SofSurfaces Inc, grants and personal fees from SorbaShock LLC, grants and personal fees from Viconic Sporting, outside the submitted work. AL is a member of an ASTM Work Group (WK38804) whose Technical Contact is the President of SATech. SATech has donated flooring materials to AL's laboratory that have formed the basis of several studies examining the biomechanical effectiveness of compliant flooring (i.e. safety flooring). He has never had (nor does he currently have) any financial links to the company.

CL is employed at the Canadian Agency for Drugs and Technologies in Health (CADTH).

JR is a member of the NIHR's HTA/EME editorial board (0.1 wte).

LF has nothing to disclose.

Additional Files

Supplementary file 1. PRISMA-P checklist.

Supplementary file 2. Medline search strategy

Figure 1. Theoretical framework of potential effect modifiers.

For peer review only - http://bmjopen.bmj.com/site/about/guidelines.xhtml

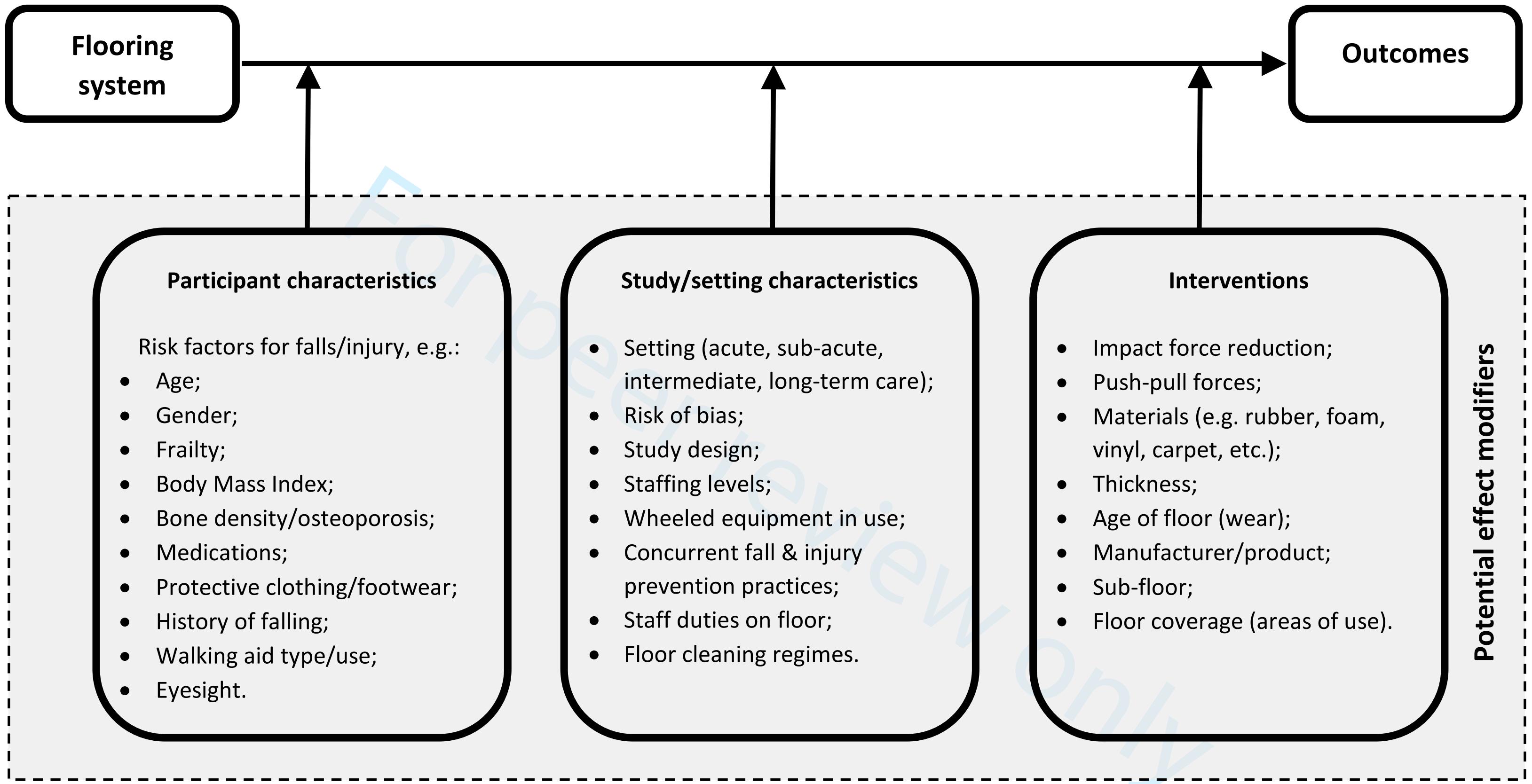
Flooring system
Outcomes
Participant characteristics
Risk factors for falls/injury, e.g.:
• Age;
• Gender;
• Frailty;
• Body Mass Index;
• Bone density/osteoporosis;
• Medications;
• Protective clothing/footwear;
• History of falling;
• Walking aid type/use;
• Eyesight.
Study/setting characteristics
• Setting (acute, sub-acute, intermediate, long-term care);
• Risk of bias;
• Study design;
• Staffing levels;
• Wheeled equipment in use;
• Concurrent fall & injury prevention practices;
• Staff duties on floor;
• Floor cleaning regimes.
Interventions
• Impact force reduction;
• Push-pull forces;
• Materials (e.g. rubber, foam, vinyl, carpet, etc.);
• Thickness;
• Age of floor (wear);
• Manufacturer/product;
• Sub-floor;
• Floor coverage (areas of use).
Potential effect modifiers

PRISMA- P 2015 Checklist

This checklist has been adapted from the operationalized checklist available from: http://www.prisma-statement.org/Extensions/Protocols.aspx which is based on Table 3 in Moher et al. Syst Rev. 2015;4(1):1. doi: 10.1186/2046-4053-4-1

Section/topic	#	Checklist item	Information reported		Page number(s)
			Yes	No	
**ADMINISTRATIVE INFORMATION**					
**Title**					
Identification	1a	Identify the report as a protocol of a systematic review	☒	☐	1
Update	1b	If the protocol is for an update of a previous systematic review, identify as such	☐	☒	n/a
**Registration**	2	If registered, provide the name of the registry (e.g., PROSPERO) and registration number in the Abstract	☒	☐	2
**Authors**					
Contact	3a	Provide name, institutional affiliation, and e-mail address of all protocol authors; provide physical mailing address of corresponding author	☒	☐	1
Contributions	3b	Describe contributions of protocol authors and identify the guarantor of the review	☒	☐	22
**Amendments**	4	If the protocol represents an amendment of a previously completed or published protocol, identify as such and list changes; otherwise, state plan for documenting important protocol amendments	☒	☐	5
**Support**					
Sources	5a	Indicate sources of financial or other support for the review	☒	☐	22
Sponsor	5b	Provide name for the review funder and/or sponsor	☒	☐	22
Role of sponsor/funder	5c	Describe roles of funder(s), sponsor(s), and/or institution(s), if any, in developing the protocol	☒	☐	22
**INTRODUCTION**					
**Rationale**	6	Describe the rationale for the review in the context of what is already known	☒	☐	4
**Objectives**	7	Provide an explicit statement of the question(s) the review will address with reference to participants, interventions, comparators, and outcomes (PICO)	☒	☐	5
**METHODS**					

For peer review only

Section/topic	#	Checklist item	Information reported		Page number(s)
			Yes	No	
**Eligibility criteria**	8	Specify the study characteristics (e.g., PICO, study design, setting, time frame) and report characteristics (e.g., years considered, language, publication status) to be used as criteria for eligibility for the review	☒	☐	5-7
**Information sources**	9	Describe all intended information sources (e.g., electronic databases, contact with study authors, trial registers, or other grey literature sources) with planned dates of coverage	☒	☐	7-8
**Search strategy**	10	Present draft of search strategy to be used for at least one electronic database, including planned limits, such that it could be repeated	☒	☐	Supplementary file 2
STUDY RECORDS					
Data management	11a	Describe the mechanism(s) that will be used to manage records and data throughout the review	☒	☐	8
Selection process	11b	State the process that will be used for selecting studies (e.g., two independent reviewers) through each phase of the review (i.e., screening, eligibility, and inclusion in meta-analysis)	☒	☐	9
Data collection process	11c	Describe planned method of extracting data from reports (e.g., piloting forms, done independently, in duplicate), any processes for obtaining and confirming data from investigators	☒	☐	9,11
**Data items**	12	List and define all variables for which data will be sought (e.g., PICO items, funding sources), any pre-planned data assumptions and simplifications	☒	☐	9
**Outcomes and prioritization**	13	List and define all outcomes for which data will be sought, including prioritization of main and additional outcomes, with rationale	☒	☐	9,10
**Risk of bias in individual studies**	14	Describe anticipated methods for assessing risk of bias of individual studies, including whether this will be done at the outcome or study level, or both; state how this information will be used in data synthesis	☒	☐	10 (quantitative),12 (qualitative)
DATA					
**Synthesis**	15a	Describe criteria under which study data will be quantitatively synthesized	☒	☐	11-12
	15b	If data are appropriate for quantitative synthesis, describe planned summary measures, methods of handling data, and methods of combining data from studies, including any planned exploration of consistency (e.g., I^2, Kendall's tau)	☒	☐	11-12
	15c	Describe any proposed additional analyses (e.g., sensitivity or subgroup analyses, meta-regression)	☒	☐	12
	15d	If quantitative synthesis is not appropriate, describe the type of summary planned	☒	☐	12, 13
**Meta-bias(es)**	16	Specify any planned assessment of meta-bias(es) (e.g., publication bias across studies, selective reporting within studies)	☒	☐	10, 11

For peer review only

Section/topic	#	Checklist item	Information reported		Page number(s)
			Yes	No	
**Confidence in cumulative evidence**	17	Describe how the strength of the body of evidence will be assessed (e.g., GRADE)	☒	☐	13,14

For peer review only

Medline search strategy

Search line	Terms
1	MH "Wounds and Injuries+
2	MH "Accidental Falls/PC"
3	MH "Hip Fractures+/PC"
4	falls
5	faller$
6	S1 OR S2 OR S3 OR S4 OR S5
7	MH "Aged+"
8	MH "Middle Aged"
9	Older
10	Senior$
11	elderly
12	S7 OR S8 OR S9 OR S10 OR S11
13	S6 AND S12
14	MH "Residential Facilities+"
15	MH "Long-Term Care"
16	MH "Institutionalization"
17	MH "Hospitalization"
18	MH "Subacute Care"
19	MH "Hospitals+"
20	MH "Hospital Units"
21	MH "Rehabilitation Centers"
22	MH "Inpatients"
23	MH "Geriatric Assessment"
24	("long stay" or "long term" or "acute" or "sub-acute" or "subacute" or "residential" or "hospital") N3 (care or ward# or hospital)
25	(rehabilitation or geriatric) N1 (ward# or hospital# or unit# or department#)
26	hostel$ or nursing home$
27	inpatient
28	resident$
29	institution$
30	S14 OR S15 OR S16 OR S17 OR S18 OR S19 OR S20 OR S21 OR S22 OR S23 OR S24 OR S25 OR S26 OR S27 OR S28 OR S29
31	S13 and S30
32	floor* NOT (pelvic floor OR sinus OR mouth)
33	carpet*
34	ground surface$
35	smartcell*
36	tarkett
37	softile
38	sorbashock
39	forbo
40	kradal
41	noraplan
42	MH "Floors and Floorcoverings"
43	S32 OR S33 OR S34 OR S35 OR S36 OR S37 OR S38 OR S39 OR S40 OR S41 OR S42
44	S31 AND S43
45	MH "Animals+"
46	MH "Humans"
47	S45 NOT S46
48	S44 NOT S47
49	S44 NOT S47<br>Limiters - Date of Publication: 20160501-

For peer review only
